# Delay reduction in MTC using SDN based offloading in Fog computing

**DOI:** 10.1371/journal.pone.0286483

**Published:** 2023-05-30

**Authors:** Zahra Arefian, Mohammad Reza Khayyambashi, Naser Movahhedinia

**Affiliations:** Department of Computing Architecture, Faculty of Computer Engineering, University of Isfahan, Isfahan, Iran; University of Jeddah, SAUDI ARABIA

## Abstract

Fog computing (FC) brings a Cloud close to users and improves the quality of service and delay services. In this article, the convergence of FC and Software-Defined-Networking (SDN) has been proposed to implement complicated mechanisms of resource management. SDN has suited the practical standard for FC systems. The priority and differential flow space allocation have been applied to arrange this framework for the heterogeneous request in Machine-Type-Communications. The delay-sensitive flows are assigned to a configuration of priority queues on each Fog. Due to limited resources in the Fog, a promising solution is offloading flows to other Fogs through a decision-based SDN controller. The flow-based Fog nodes have been modeled according to the queueing theory, where polling priority algorithms have been applied to service the flows and to reduce the starvation problem in a multi-queueing model. It is observed that the percentage of delay-sensitive processed flows, the network consumption, and the average service time in the proposed mechanism are improved by about 80%, 65%, and 60%, respectively, compared to traditional Cloud computing. Therefore, the delay reductions based on the types of flows and task offloading is proposed.

## 1. Introduction

Machine-Type-Communications (MTC) are of significant concern in both the academic and industrial world [1, 2]. MTC networks can connect devices for daily life uses such as healthcare, emergency responses, transportation, industrial automation, smart city, smart home, finance, and energy [3–5]. In MTC, billions of machines generate a massive volume of data, and they are connected to the core network infrastructure [3]. MTC services have various requirements, including quality of service (QoS), load balancing, and overload techniques [5, 6]. Cloud Computing has denoted the essential execution for MTC applications. MTC requests face many challenges, including the lack of global mobility, location-aware applications due to the several machine types of MTC, heavy load on Cloud servers, and disability in fully conforming with delay-sensitive (real-time processing) [7, 8]. Fog computing (FC) can help organizations reduce latency issues. Cisco proposed FC in 2012, which reduces the service delay and the network traffic and network congestion by processing the requests on Fog nodes instead of forwarding them to the Cloud data center from the core network [9–14]. If requests require permanent storage or extensive analyses, they are sent to the Cloud. FC is not to be substituted but is a complement to Cloud computing to ease the bandwidth burden and reduce the latency [15, 16]. In the heterogeneity MTC requests, integration of the Fog technology using virtualization is isolated, scalable, and flexible for computation and improving efficiency in networks [17, 18].

Traditional Fog networks face challenges such as centralized control, programmability, load balancing, and manageability [19, 20]. Software-Defined-Networking (SDN) can be used to achieve effective collaboration among Fog nodes [21, 22]. SDN provides programming flexibility of the network by improving the rule policies in its centralized SDN controller with the network global view [23]. An SDN controller manages the network by gathering all information on every network device, updating the load balancing policy, and overseeing the network traffics in a dynamic manner [24, 25].

The FC context proposes low-latency to the MTC flows due to a computation flow offloading decision and resource allocation scheme. The offloading strategies are based on static or dynamic schemes. The static scheme selects a specified offloading node for each Fog node. In contrast, the dynamic scheme selects an optimal offloading Fog node from the available Fog nodes in the system based on their queue conditions, strategies, and certain real-time conditions [21]. The dynamic method can be distributed. In the distributed approach, the offloading node is selected by the overloaded node based on their shared information with other Fog nodes. The local Fog can offload the computation flows to other Fogs. When Fogs offload their flows, they have little information about the other Fog networks, path delay, and traffic load that could be accessed.

**Fig 1:**
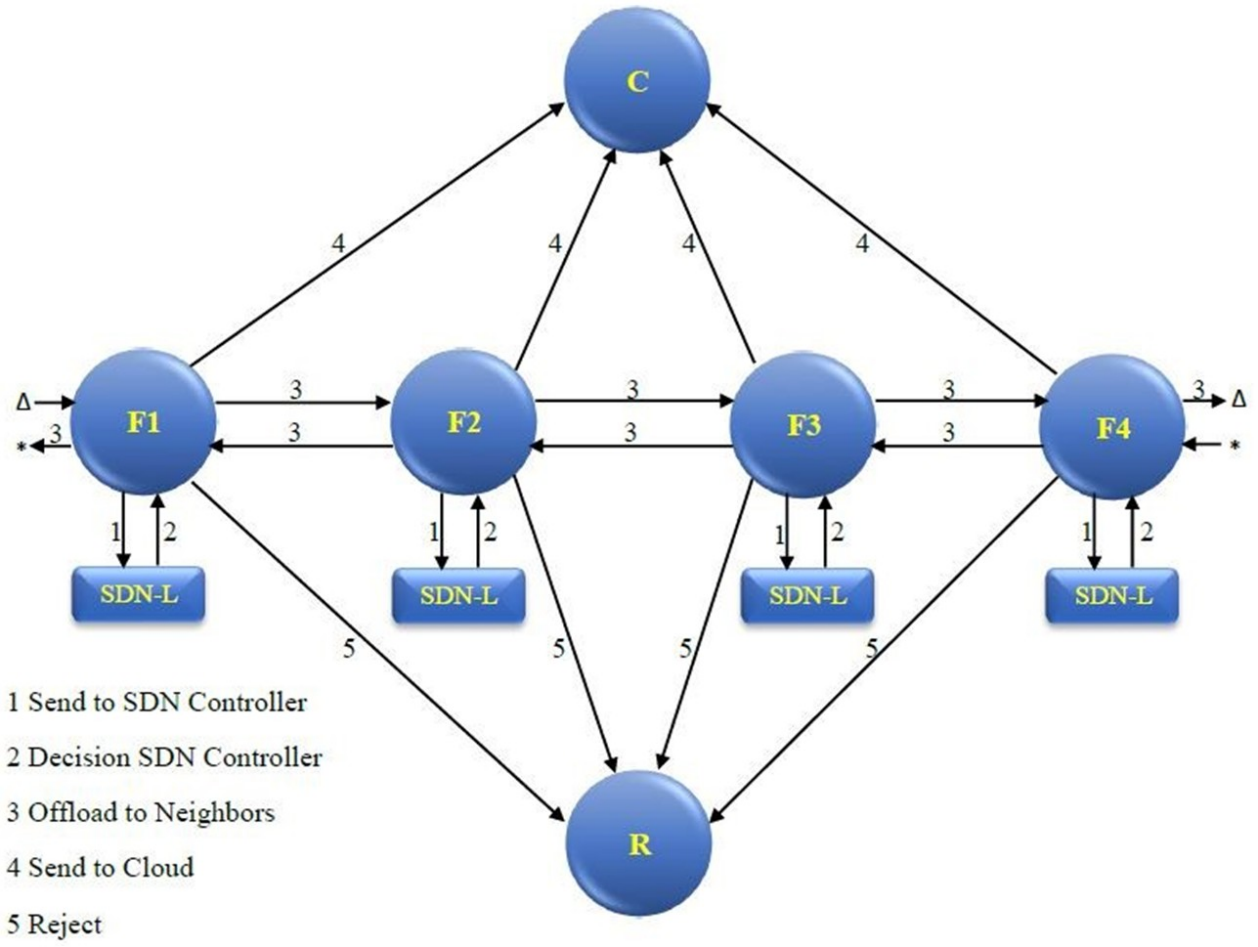
The request process procedure.

Consequently, the network information collected through the SDN controller by applying the southbound APIs conforming to its network global view, makes flow offloading decisions possible [26]. There is an SDN controller in each Fog node. Based on the decision drawn by the SDN controller, the flow is processed locally or is offloaded on its neighboring Fogs for processing. The SDN controller devises a table of neighbors and decides whether to offload their flows that the local Fog cannot process. The SDN controller provides computing services with high performance to delay-sensitive flows through offloading and FC (task placement problem) [9, 27]. A dynamic offloading mechanism is proposed in SDN-based FC systems. An example of how to process or reject each flow in this proposed framework is shown in Fig 1. The proposed framework includes four patches of Fog, shown in F1, F2, F3, and F4 states. Each Fog node has two neighbors. Each flow that cannot be processed locally, the Fog nodes are sent to the local SDN controller (No. 1). Based on the decision drawn by the SDN controller (No. 2), the flows can be offloaded to the related neighbors (No. 3) or processed in Cloud (No. 4), as shown in the C state. Alternatively, flows can be rejected (No. 5), as shown in the R state.

Different QoS requirements in massive MTC traffic are usually accompanied by heavy overhead in the SDN control plane, including data distribution and monitoring in the local Fog, offloaded data on neighboring Fogs, and Cloud computing [5, 28]. An optimization problem presents delay-sensitive utilization of available Fog resources based on VRs. Due to the difference in the priority of flows sent by MTC machines, queues of different priorities are formed behind the Fog nodes. Polling algorithms are applied to service flows in the queue and remove the starvation problem for low priority flows. Polling systems are priority-based scheduling schemes in a multi queueing model [29, 30].

Most of the available studies propose FC based on the architectural FC and delay reductions in FC, while a few are based on delay reductions in FC depending on the types of flows and task offloading. In some studies, a flexible construction for MTC flows is proposed based on FC and SDN by applying a global view that highlights delay reduction based on queuing priorities and deadlines.

The main research question is formulated as follows: "Can better performance be achieved by offloading machine requests to neighboring nodes in the Fog layer?". In brief, the unique contributions made in this work than the existing state-of-art research are:

- A novel framework based on a task offloading scheme in SDN for MTC machines in Fog networks.
- The following two main points are typically involved in the offloading method: where should flows be offloaded to directly affect the system’s performance, and how should this be done.
- A new model of MTC architecture is proposed for manageability and low latency.
- Priority and differential flow space allocation designs are utilized to attend to critical and urgent flows. SDN focuses on fairness among the normal flows by applying programmability.
- The delay minimization problem is formulated through the queueing offloading decisions.

The rest of this article is organized as follows: Section 2 provides an overview of related works. Section 3 describes the considered system model, and Section 4 explains the simulation experiment. Finally, Section 5 concludes the article and provides future directions for this work.

## 2. Related work

In this section, the previous papers have discussed the task offloading problem in Fog as well as the possible additional contributions that have concerned significant attention from researchers.

Du et al. [31] provided methods for offloading decisions and resource allocation in a Fog system. The optimization problem was formulated as a mixed integer non-linear programming problem. In [32], a novel three-tier architecture was proposed, where the task response time of each user was minimized through offloading strategies using a generalized Nash equilibrium based on queueing theory. In [33], the authors proposed Volunteer Supported FC (VSFC), which minimized inherent communication delays, energy consumption, and network usage. VSFC reduces the cost of maintaining high-performance computing used for delay-sensitive IoT applications. For IoT-Fog-Cloud applications, Yousefpour et al. [4, 34] proposed a general framework to support low latency services, fast response time, and real-time requests by offloading the Fog policy to improve QoS. In [26], the authors proposed the dynamic optimal task offloading problem in software-defined access networks, which offers low latency and flexible computation. Chen et al. [35] proposed an offloading algorithm for dynamic computation to the edge of the network to minimize the offloading cost while avoiding a significant load on the network. The theoretical analysis demonstrated that this algorithm could optimize the offloading cost and bound queue length. For Fog-enabled IoT networks, Wang et al. [9] proposed a latency-minimum offloading decision and resource allocation scheme to alleviate the burden of core network communication. In [36], the authors proposed an offloading method to balance Fog load. Their approach reduces latency by using the Fog-to-Fog collaboration model to distribute requests and distinguish between IoT heavy requests and light requests. A hybrid normal data propagation framework applies SDN. To reduce delays and enhance performance in delay-sensitive tasks [27], the authors investigated the offloading problem for requests in the software-defined ultra-dense networks. The task of offloading at the edge of the Cloud or processing locally was formulated by a mixed-integer nonlinear program. To enhance various performance metrics [37], Alnoman et al. provided a holistic view and effective solutions to communication and computing challenges in edge IoT systems to tackle different system-level aspects such as computing with learning features, delay, scheduling, energy consumption, and resource management. An analytical model for data centers has been proposed in [38] as a simple offloading strategy under heavy loads in FC. A new delay-dependent priority was proposed in [39] that aware offloading strategy for processing the tasks, scheduling, and minimizing the starvation problem of low-priority tasks, stating that the offloading strategy and multilevel-feedback queue could help meet the deadline due to the resource requirements and communication time. In [14], the authors proposed an architecture that uses a dynamically offloading threshold in delay-sensitive vehicular traffic. This method used DEC and DTS algorithms at the Fog layer to solve the total delay, minimize energy consumption, and improve throughput. Li et al. [40] proposed a three-layer hierarchy scheme for the SDN controller framework to reduce the delay between the SDN controller and the switch. To enhance the performance of the Internet of Vehicles, they used Mobile-edge computing and SDN. In [41], the authors proposed an Energy-effective task offloading strategy in the system. They formulated the delay problem of task allocation and minimum energy consumption. In [42], the authors proposed a PSO algorithm based on LPSO algorithms. They suggested balancing transmission, computing, and computational energy to minimize energy consumption and task delay. A way to minimize energy consumption and delay of tasks was obtained by them.

A traffic-aware load-balancing scheme for M2M networks was proposed in [5], which applied SDN switches to update the flow table and meet various QoS M2M traffic requirements in SDN by dynamically rerouting traffic and identifying immediate traffic. In [34, 43], a scheme was proposed to reduce delay and minimize service delay for Fog devices. Hakiri et al. [43] proposed an SDN controller Fog architecture to provide load balancing and facilitate traffic engineering among Fog devices. A secure distributed Fog node architecture was proposed in [44] to reduce delay based on blockchain technology that improved performance. It applied SDN to high-performance computing and performed efficiently. In [13], the authors proposed a method for real-time IoT applications with QoS requirements in edge computing environments. This method revealed a novel research view about QoS dynamic management. This study integrated the MEC standard architecture of [45] to establish configuration management with a network function virtualization (NFV) platform and utilized a flow control mechanism for 5G networks. A CF-CloudOrch architecture was noted in [8], which guaranteed distributed networks’ simple management and high performance. It solved many problems, including scheduling, load balancing, scalability, security, and flexibility. In [1], a framework was proposed for M2M communications that converged wireless virtualization of cellular networks’ software according to QoS requirements and different functions. Furthermore, a control loop was developed to dynamically allocate the virtual resource block counts to improve QoS.

An improved firefly algorithm was noted in [46], which guaranteed workflow scheduling in Cloud edge with shorter response time and less network bandwidth consumption. They incorporated a quasi-reflection-based learning method and genetic operators. In [47], a framework was proposed to address resource-related constraints such as scheduling and load balancing. An improved version of the min-min algorithm was proposed, which considers energy, makespan, and cost in a heterogeneous environment. In [48], the authors proposed a reinforcement learning algorithm for Fog scheduling to accomplish crucially significant challenges such as minimizing energy consumption, load balancing, and scheduling requests.

In [49], the authors proposed a bid prediction mechanism to optimize computation offloading using auction theory. The mechanism was based on Q-learning, where nodes bid to offload tasks on their upstream node, and the winning node offloads the task. The proposed method consumed less energy, reduced execution time, and saved network resources compared to traditional techniques. In [50], the authors proposed an incentive-compatible offloading scheme to minimize latency and energy consumption for user tasks using an auction algorithm in the Fog computing environment and the Cloud layer. They formulated the problem using queuing theory. To minimize time delay and power consumption in Fog computing for IoT devices, [51] the authors formulated the problem of joint optimization using the Bees algorithm and the genetic algorithm. They improved the solution quality with a minimax differential evolution. The NP-hard problem was solved using several optimization methods. In [19], the authors proposed a converged SDN and Fog computing that employed differential flow space allocation for heterogeneous IoT applications per flow classes to satisfy priority-based quality of service requirements.

In [52], the authors proposed an AI-based task offloading and resource distribution mechanism for reconfigurable IoV Networks. It provides reliable and fast communication in dynamic environments by using intelligent controllers to optimize resource utilization and reduce delays.

**Fig 2:**
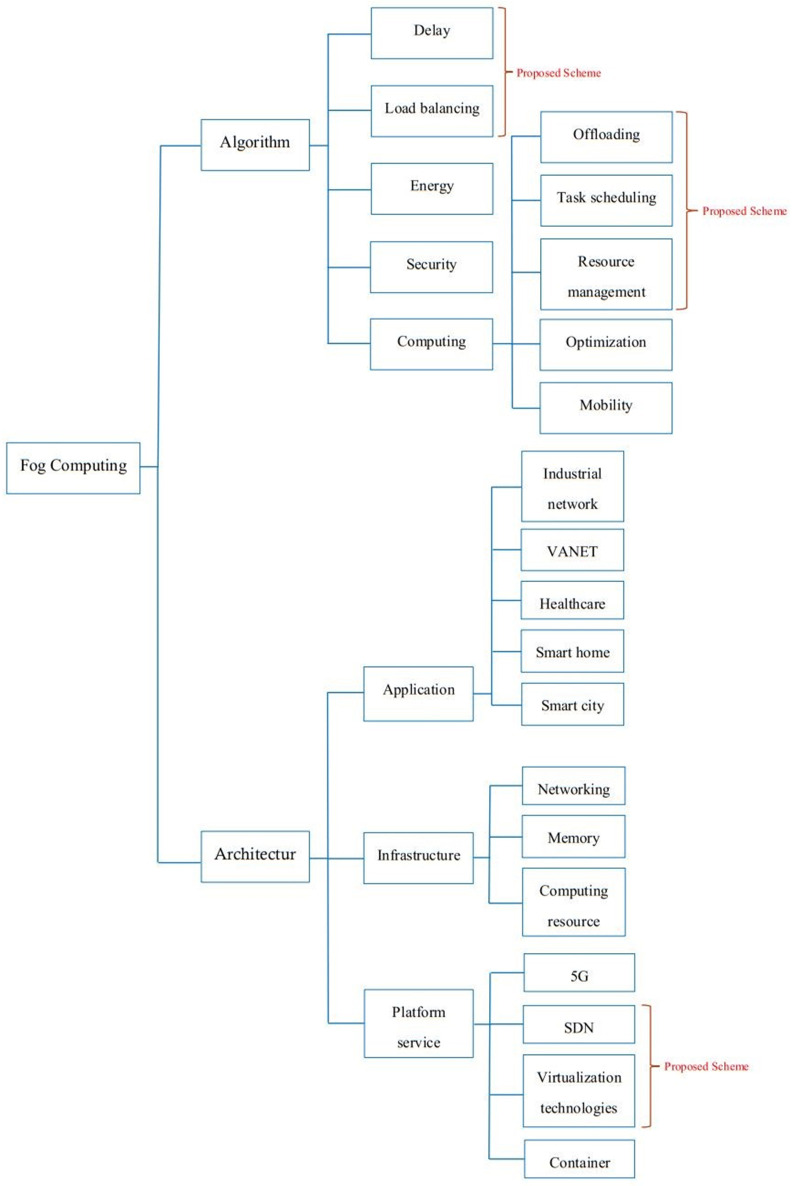
Taxonomy of Fog computing.

According to the review, the task offloading problem in FC is assessed to minimize MTC total delay, which is a fundamentally different major technology in solving the same problem. The important drawbacks of the current methods are the lack of serious considerations for resource management for different types of flows, reducing the number of dropped flows, and removing the starvation problems based on the schedule for lower priority flows. The rest of this article discusses the proposed offloading FC framework for reducing the delayed scheme due to the problems mentioned. Table 1 compares the most important existing reviewed research and proposed scheme. Additionally, Fig 2 describes the taxonomy of Fog Computing based on architecture and algorithm.

**Table 1:** Comparison of the most important related works.

Ref.	Year	Feature	SDN	FC	Type of Request	Offloading strategy	Multiple queues	Reduce the starvation	Response time
[4]	2018	improve QoS, reduce the service delay.	✘	**✓**	**✓**	**✓**	**✓**	✘	**✓**
[5]	2018	traffic-aware load balancing scheme, control the network by SDN.	**✓**	✘	**✓**	✘	✘	✘	**✓**
[9]	2020	resource allocation, genetic simulated, optimal latency-minimum offloading decision algorithm.	✘	**✓**	**✓**	**✓**	✘	✘	**✓**
[14]	2021	an optimisation dynamic offloading scheme for minimising overall delay, improving throughput, and minimising energy consumption at the Fog layer.	✘	**✓**	**✓**	**✓**	✘	✘	**✓**
[27]	2018	Task offloading optimization problem in ultra-dense network to minimize the delay and saving the battery life, efficient scheme with task placement and resource allocation.	**✓**	**✓**	**✓**	✘	✘	✘	**✓**
[33]	2020	reduction in the maintaining high-performance computing cost, minimize energy consumption and network usage and communication delays.	✘	**✓**	✘	✘	✘	✘	**✓**
[39]	2019	a priority task offloading strategy for scheduling and processing the tasks, minimizes the starvation, multilevel-feedback queue, reduces the delay-sensitive tasks time.	✘	**✓**	**✓**	**✓**	✘	**✓**	**✓**
[40]	2021	enhancing the performance of IoV, minimizing the delay between the switch and the controller, the optimal location of controllers subject to load balance index and buffer size.	**✓**	**✓**	✘	✘	✘	✘	**✓**
[41]	2022	strategy of Energy-effective in the system, delay-aware task allocation problem.	✘	**✓**	✘	✘	✘	✘	**✓**
[42]	2022	reducing the delay of delay-sensitive tasks, minimum energy consumption, a heuristic particle swarm optimization (PSO) algorithm	✘	**✓**	**✓**	✘	✘	✘	**✓**
[43]	2017	lower latency and efficient load balancing to offload the network load by enabling programmable Fog routers.	**✓**	**✓**	✘	✘	✘	✘	**✓**
[44]	2017	novel blockchain distributed cloud architecture with a SDN, low-cost, secure, on-demand access, high-performance computing.	**✓**	**✓**	✘	✘	✘	✘	**✓**
[46]	2022	workflow scheduling in cloud edge, shorter response time and less network bandwidth, incorporated quasi-reflection-learning method and genetic operators.	✘	**✓**	✘	✘	✘	✘	**✓**
[47]	2022	min-min algorithm, scheduling, load, and cost in heterogeneous environment.	✘	**✓**	✘	✘	✘	✘	**✓**
[48]	2023	reinforcement learning algorithm, minimizing energy, the load balancing, and scheduling requests.	✘	**✓**	**✓**	✘	✘	✘	**✓**
[49]	2023	optimizing computation offloading using Q-learning, consumed less energy, reduced execution time, and saved network resources.	✘	✓	✘	✓	✘	✘	✓
[50]	2021	incentive-compatible offloading scheme to minimize latency and energy consumption.	✘	✓	✘	✘	✘	✘	✓
[51]	2021	minimized time delay and power consumption, optimization using the Bees and genetic algorithms, improved solution quality with a minimax differential evolution.	✘	✓	✘	✘	✘	✘	✓
[19]	2018	converged SDN and FC that employed differential flow space allocation for heterogeneous IoT applications per flow classes.	✓	✓	✓	✘	✓	✘	✓
[52]	2020	task offloading and resource distribution in AI for IoV, reliable and fast communication, optimize resource and reduce delays.	✓	✓	✘	✓F	✘	✘	✓
proposed		A new framework based on a task offloading scheme in SDN for MTC machines in Fog networks, manageability and low latency, priority and differential flow space, queueing offloading decisions, load balancing, minimizes the starvation for low priority flows using polling algorithm.	**✓**	**✓**	**✓**	**✓**	**✓**	**✓**	**✓**

## 3. Proposed scheme and problem formulation

Flows are generated by the MTC machines and sent through OpenFlow switches located at the edge of the data center. Depending on the MTC machine that generated them, their type is determined by the SDN controller. The flows can be either delay-sensitive or non-delay-sensitive, and they can be complex or simple, requiring heavy or light processing resources. Therefore, the SDN controller determines the type of each flow based on the type of sending MTC machine. This scheme has four types of flows, which are specified in the header.

1. Delay-sensitive Lightweight (DL),
2. Delay-sensitive Heavy (DH),
3. Non-Delay-sensitive Lightweight (NDL),
4. Non-Delay-sensitive Heavy (NDH).

### 3.1. Network model

**Fig 3:**
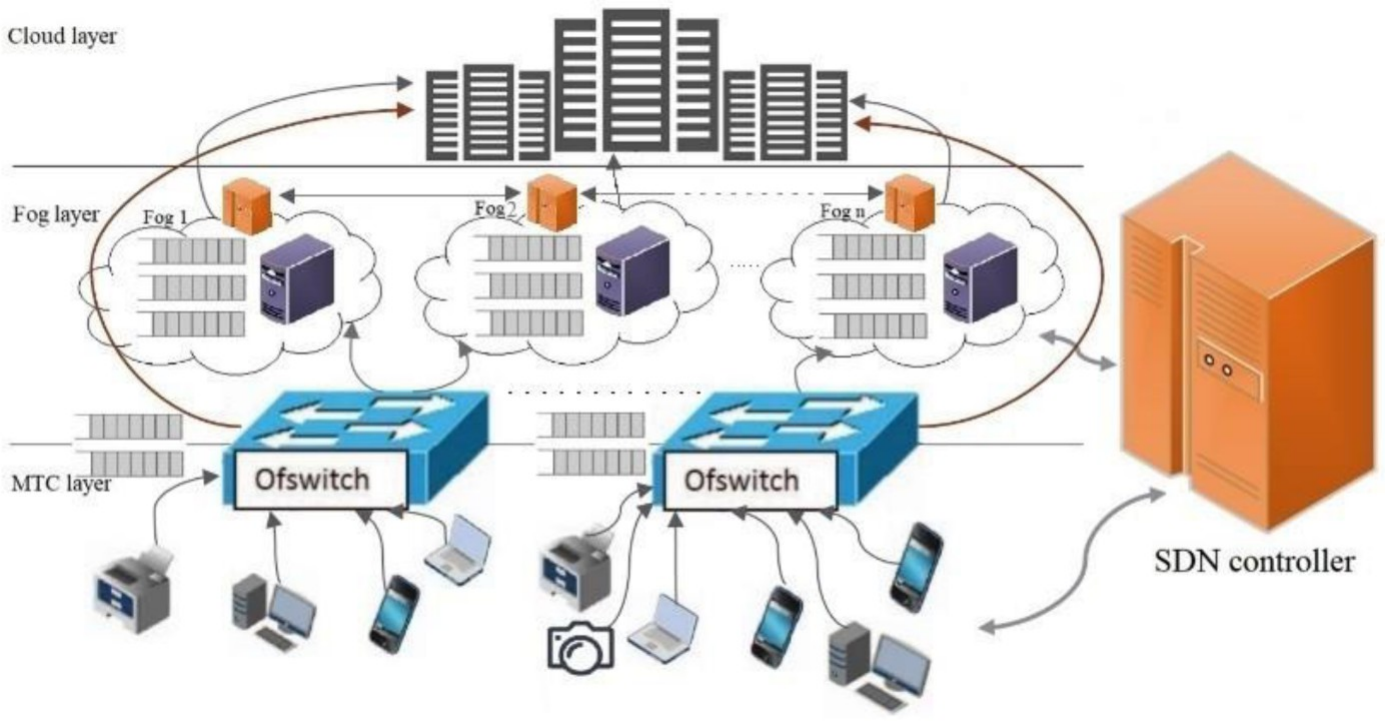
Hierarchical Fog cloud model for MTC machine.

A hierarchical Fog-Cloud scenario is illustrated in Fig 3. This architecture defines three layers horizontally: MTC layer, Fog layer, and Cloud layer. Additionally, a main SND controller layer is illustrated. An SND layer is defined, which connects with MTC layer and Fog layer, and manages the entire network and MTC requests. The SDN controller can be applied to manage the communications in Cloud or Fog environments. The networks are constructed by defining Machine to Fog Communications (M2F), Fog to Cloud Communications (F2C), Machine to Cloud Communications (M2C), and Fog to Fog Communications (F2F). The edge switch sends each flow to the main SDN controller to identify its type. Then, all flows must be sent to Fog or Cloud for processing. If the type of flow is DL, DH, or NDL, it is sent to Fog and if the type is NDH, it is directly sent to Cloud. Thus, they are communications of type M2F and M2C, respectively. There is a SDN controller in each Fog node. In the Fog layer, the SDN controller architecture is the flat model. The SDN controllers connect via East/Westbound API to their neighbors. Therefore, they can contact and notify each other directly, making them communications of type F2F.

This study considers a group of Fog nodes, where the set of *n* Fog networks is denoted as **F *=* {*F***_**1**_, ***F***_**2**_,**…, *F***_***n***_***}***. Each Fog network *F*_*i*_ consists of *m* VRs, defined as **F**_**i**_ ***=* {*f***_***i1***_, ***f***_***i2***_,**…, *f***_***im***_**}**. The total number of MTC devices is denoted as *M*, which includes *r* flows represented by **R *=* {*1*, *2*, *…*, *r*}**, and a Cloud server denoted as *S*. For any flows, *(X*, *T*_*pro*_*)* is used where *X* represents the input data size, and *T*_*pro*_ depicts the processing delay.

### 3.2. Delay model

If the flow is NDL, it can be processed in the Cloud or the Fog, as shown in Eq (1). Here, *ω*_*i*_*∈{0*,*1}* is the fairness parameter, when *ω*_*i*_ *= 0* indicates that the flow is processed to the local Fog and *ω*_*i*_ = 1 indicates that it is processed in the Cloud server. The value of *ω*_*i*_ depends on where the flow is processed. T_t-Fog-NDL-i_ and T_t-Cloud-NDL-i_ represent total delay for NDL flows in Fog and Cloud, respectively. More details about this equation are discussed in the following section. The key notations have been summarized in Table 2.


$${\text{T}}_{\text{Total-NDL}}=\left(1-\omega_{\text{i}}\right)\;{\text{T}}_{\text{t-Fog-NDL-i}}+\left(\omega_{\text{i}}\right)\;{\text{T}}_{\text{t-Cloud-NDL-i}}$$ (1)

**Table 2:** Table of notations.

Notations	Definitions
*F*, *R*	Set of Fog nodes and Set of flows
*DL*, *DH*, *NDL*, *NDH*	Type of requests
*M2F*, *F2C*, *M2C*, *F2F*	Type of flow communication
*ω*_*i*_	Task processing or rejecting the decision of device i
*θ*_*i*_	Task sending to Cloud or local Fog decision of device i
*δ*_*i*_	Task sending to Cloud or local Fog decision of device i
*ζ*_*i*_	Task offloading decision of device i
*n*,*m*	The count of Fog networks and VRs on Fog
*T*_*x*_	Different types of delays such as transmission, propagation, and etc
*D_V*	Data volume
*B*	Bandwidth
*Dis_Fog*	Distance to resource
*F-S*	Flow speed
*P*_*i*_	Probability service in queue i
*r*_*i*_	Weight of priority polling queue

In Eqs (2), (3) and (4), the offloading decision for delay-sensitive flows *i* is marked according to the SDN controller management as *θ*_*i*_*∈{0*,*1}* is the fairness parameter, when *θ*_*i*_ *= 0*, it means that the ith flow is processed; otherwise, it is rejected. T_t-Process-DL-i_ and T_Reject-i_ are total delay to process DL flows in Fog or reject them, respectively. For this type of flow, *δ*_*i*_ *∈{0*,*1}* is defined. *δ*_*i*_ *= 0* means that the flow i is processed in Fog resource and *δ*_*i*_ *= 1* means that the flow i is processed to the Cloud server. T_t-Fog-DL-i_ and T_t-Cloud-DL-i_ are total delay for DL flows in Fog and Cloud, respectively. If a flow is processed in Fog resource, it may be processed in local Fog resource or is offloaded to the neighboring Fog, *ζ*_*i*_*∈{0*,*1}* is the fairness parameter, which *ζ*_*i*_ *= 0* means that the flow i is processed in local Fog resource, and *ζ*_*i*_ *= 1* means that it is offloaded to other Fogs. T_t-LocalFog-DL-i_ and T_t-Offload-DL-i_ are total delay for DL flows in local Fog or neighboring Fog nodes, respectively (more details in continued section). The following explanations are obtained for DL, *T*_*Total-DH*_ could be derived similarly.


$${\text{T}}_{\text{Total-DL-i}}=\left(1-\theta_{\text{i}}\right)\;{\text{T}}_{\text{t-Process-DL-i}}+\left(\theta_{\text{i}}\right)\;{\text{T}}_{\text{Reject-i}}$$ (2)

$${\text{T}}_{\text{t-process-DL-i}}=\left(1-\delta_{\text{i}}\right)\;{\text{T}}_{\text{t-Fog-DL-i}}+\left(\delta_{\text{i}}\right)\;{\text{T}}_{\text{t-Cloud-DL-i}}$$ (3)

$${\text{T}}_{\text{t-Fog-DL-i}}=\left(1-\zeta_{\text{i}}\right)\;{\text{T}}_{\text{t-LocalFog-DL-i}}+\left(\zeta_{\text{i}}\right)\;{\text{T}}_{\text{t-Offload-DL-i}}$$ (4)

The proposed model is a network that is managed by the SDN controller. When a flow is sent from the first layer to the second layer, it is transmitted via edge OpenFlow switches located in the data panel. Each OpenFlow switch has a complete flow table that determines the path of MTC machine’s flows and transmits them. The OpenFlow switch decides which Fog node to send the flow according to the flow table. In other words, switches can handle links by applying flow headers, flow status and flow table. Depending on the priority of the flows, there are two queues of switches. If the type of flow is delay-sensitive, it is sent to the first queue by the SDN controller, and if the type of flow is NDL, it is sent to the second queue, as illustrated in Fig 3 as shown in Q_1_ and Q_2_, respectively. Thus, the differential queue of heterogeneous type of flow is inevitable at the OpenFlow switch, which is supported and managed by the global SDN controller. If there are k switches for each Fog, M/M/k queueing theory can be applied. The delay for each flow at this stage can be calculated using the following equations.

In Eq (5), *T*_*1i*_ represents switch delay and total delays until *i*th flow reaches the Fog layer. Section 3.3 provides a more detailed explanation of the queueing theory (*T*_*QSi*_). *T*_*Transi*_ and *T*_*Propi*_ represent Transmission delay and Propagation delay, respectively. *T*_*SDNi*_ is a total Round trip flow time from the edge switch to the SDN controller and additional processing in the SDN controller. *T*_*Fogi*_ is queue processing time on path switches to reach the Fog with a minimum service rate. In Eq (6), *D-V*_*i*_ and *B*_*i*_ represent data volume and bandwidth. *Dis_Fog* and *F-S* represent the distance to Fog resource and flow speed, respectively. In Eq (7), *T*_*PropSDNi*_ indicates Propagation delay to SDN controller. *T*_*ProSDNi*_ is used to measure the southbound communication delay from the edge OpenFlow switch to the SDN controller by using echo packets and calculating the timestamp.


$${\text{T}}_{1\text{i}}={\text{T}}_{\text{Transi}}+{\text{T}}_{\text{PropFi}}+{\text{T}}_{\text{QSi}}+{\text{T}}_{\text{SDNi}}+{\text{T}}_{\text{Fogi}}$$ (5)

$${\text{T}}_{\text{Transi}}=\frac{D\_V_{i}}{B_{i}},{\text{T}}_{\text{PropFi}}=\frac{Dis\_Fog_{i}\;}{\text{F}\_\text{S}}$$ (6)

$${\text{T}}_{\text{Fogi}}=\frac{1}{\mu_{\text{min}}-\lambda },{\text{T}}_{\text{SDNi}}=2*{\text{T}}_{\text{PropSDNi}}+{\text{T}}_{\text{ProSDNi}}$$ (7)

When flows arrive at a Fog, they need to be managed based on several parameters such as delay sensitivity, deadline, processing time, and other parameters. Each Fog has a local SDN controller that manages the flows based on the information in their headers. On each Fog SDN controller, there is a table that contains delay information about its neighboring Fog nodes. This table is used when the Fog node decides to offload a flow to another Fog node for processing. The local SDN controller updates the information in the table of a Fog node periodically. The SDN controller calculates transmission delay, propagation delay, and queueing delay to all neighboring nodes, and then selects the first suitable neighboring Fog node. consequently, the neighbor is selected based on whether it meets the deadline for sensitive flows. An example of this table is shown in Table 3, where neighbor 1 is selected based on the deadline.

**Table 3:** The delay information of neighboring Fog node 1.

Fog 1	Transmission delay	Propagation delay	Queueing delay
Neighbor 1	~0	0.53 ms	0.234ms
Neighbor 2	0.03ms	0.12 ms	1.17ms
⋮			
Neighbor n	0.09ms	0.24 ms	0.71ms

Some VRs in each Fog resource are implemented with higher efficiency. As described at the end of this subsection, the number of VRs changes dynamically in each period to increase efficiency. There are three queues behind Fog nodes, including DL flows, DH flows, and NDL flows. As mentioned before, NDH flows are sent directly to the Cloud. According to the types of flows of arriving at Fog nodes, the SDN controller optimizes the allocation of computation resources by offloading each flow and setting the queue parameters.

Suppose the type of flow *i* reaching the Fog is DL and DH. If all Fog’s VRs are considered the same, the SDN controller calculates the following Eq (8) for them, where *T*_*Deadlinei*_ and *T*_*ProFi*_ represent the point at which sensitive flow *i* must be completed and the processing delay in VRs, respectively. Note that if the flow is processed on Fog or Cloud resources, then the total delay mainly depends on the volume of flows and frequency resources. T_2i_ represents the remaining time for delay-sensitive requests based on the processing time on Fog resources and T_1i_.


$${\text{T}}_{2\text{i}}={\text{T}}_{\text{Deadlinei}}–\left({\text{T}}_{1\text{i}}+{\text{T}}_{\text{ProFi}}\right)$$ (8)

**Fig 4:**
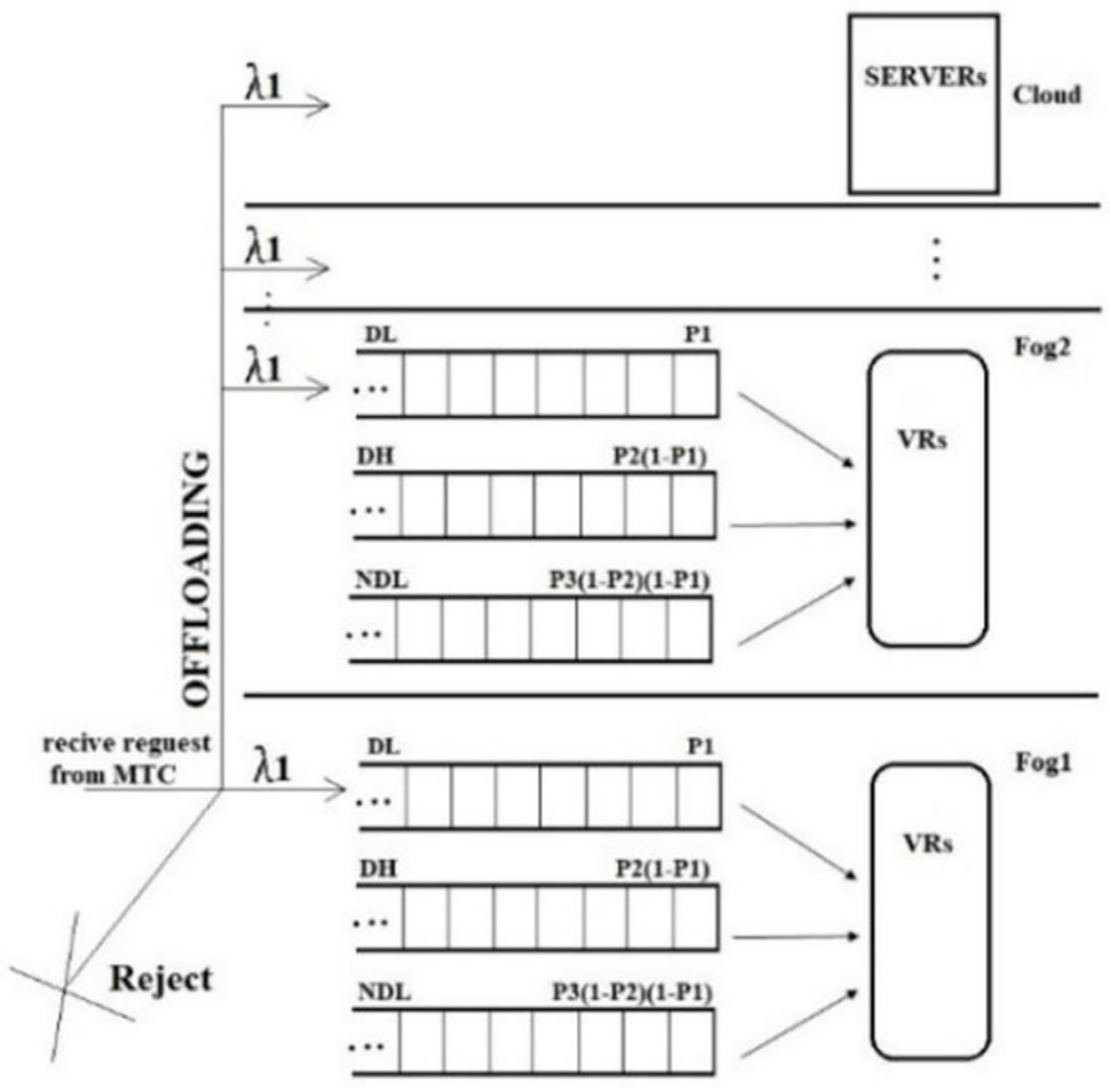
Various processing flows.

Moreover, it calculates *T*_*QRi*_ to apply queueing theory. *T*_*QRi*_ indicates the delay in the resource queue. Section 3.3 provides a more detailed explanation of the queueing theory, including the number of flows queued and the exact processing time for each flow. The queue behind the VRs is a priority queue based on the type of flows. Based on the type of flows, the SDN controller sends flows to one of the queues. The SDN controller manages computing resources and recognizes the number of available computational resources. In other words, managing the number of VRs is done through load balancing, which is explained in more detail below. When a flow is sent to the Fog SDN controller, it performs the following steps based on the available resources, their queue, and calculated equations from the table of neighbors and the delay information resulting from each Fog:

1. Accept or reject: The SDN controller needs to note that DL or DH flows should be accepted or rejected based on their deadline, while NDL flows are always accepted. The SDN controller performs the following steps to accept or reject a flow.
2. If the flow is accepted, the SDN controller schedules it for processing on either the Fog or the Cloud, according to the schedule. Eq (3) is used for this purpose.
3. Update the resource queue scheduling based on the updated TQR values, as illustrated in Fig 4.

The SDN controller calculates *T*_*QR*_, and one of the following actions is executed according to the type of flows:

1. For DL flows with specified deadlines, they are placed on the high-priority queue Q_1_, which has the highest priority. If the flows are DL, the SDN controller first tries to send them to Q_1_ for processing in the local Fog. Otherwise, it seeks to offload them to Fog neighbors and enters their Q_1_. If that is not possible, it is sent to the Cloud, and if that is not possible, it is rejected.
  In Eq (9), *T*_*ni*_ is a vector representing the *i*th flow offloading delay based on Fog to all Fog neighbors, and *n_n* is the count of its neighbors. The number of vectors is equal to the number of Fog nodes. *T*_*transi_j*_ and *T*_*propi_j*_ represent Transmission delay and Propagation delay from the local Fog to Fog neighbors j, respectively. The type of communication is F2F.
$${\text{T}}_{\text{nj}}={\text{T}}_{\text{transi\_j}}+{\text{T}}_{\text{propi\_j}}+{\text{T}}_{\text{QRi\_j}},\text{j}=1,2,\ldots ,\text{n}\_\text{n}$$ (9)
  In Eq (10), *T*_*trans_c_i*_ and *T*_*prop_c_i*_ represent the Transmission delay and Propagation delay, respectively, of the *i*th flow from the local Fog to the Cloud. *T*_*Cloud_i*_ is queue processing time on path switches to reach the Cloud with a minimum service rate. The type of communication is F2C. T_ci_ is the total delay on Cloud.
  The algorithm is summarized below.
$${\text{T}}_{\text{ci}}={\text{T}}_{\text{trans\_c\_i}}+{\text{T}}_{\text{prop\_c\_i}}+{\text{T}}_{\text{Cloud\_i}}$$ (10)
  *Algorithm process type of DL*:
   *If (T*_*QRi*_ *< T*_*2i*_*) (then ζ*_*i*_ *= δ*_*i*_ *= θ*_*i*_ *= 0* and *send to Q*_*1*_*)*
   *Else (for j = 1*:*n_n if (T*_*nj*_ *< T*_*2i*_*) (then ζ*_*i*_ *= 1 and δ*_*i*_ *= θ*_*i*_ *= 0* and *send to Fog neighbor j)*
   *Else if (T*_*ci*_ *< T*_*2i*_*) (then δ*_*i*_ *= 1* and *θ*_*i*_ *= 0* and *send to Cloud)*
   *Else (θ*_*i*_ *= 1* and *reject)*
*END*
2. If the flows are DH, the SDN controller first tries to send to Cloud; otherwise, sends it to Q_2_ for processing in the local Fog; otherwise, it seeks to offload to Fog neighbors and enter one of their Q_2_, and finally, the flow is rejected. Similar to Eqs (9) and (10), *T*_*ni*_ and *T*_*c*_ are calculated for DH (since the flow requires heavy processing, it is first attempted to be sent to the Cloud).
  *Algorithm process type of DH*:
   *If (T*_*ci*_ *< T*_*2i*_*) (then δ*_*i*_ *= 1 and θ*_*i*_ *= 0* and *send to Cloud)*
   *Else if (T*_*QRi*_ *< T*_*2i*_*) (then ζ*_*i*_ *= δ*_*i*_ *= θ*_*i*_ *= 0* and *send to Q*_*1*_*)*
   *Else (for j = 1*:*n_n if (T*_*nj*_ *< T*_*2*_*) (then ζ*_*i*_ *= 1 and δ*_*i*_ *= θ*_*i*_ *= 0 and send to Fog neighbor j))*
   *Else (θ*_*i*_ *= 1* and *reject)*
*END*
3. If the ith flow is NDL, Eq (11) calculates *T*_*4i*_ and *T*_*5i*_, which represent the total delay for NDL flows in Fog and Cloud, respectively.
$${\text{T}}_{4\text{i}}={\text{T}}_{1\text{i}}+{\text{T}}_{\text{proFi}}+{\text{T}}_{\text{QRi}},{\text{T}}_{5\text{i}}={\text{T}}_{1\text{i}}+{\text{T}}_{\text{proCi}}+{\text{T}}_{\text{propCi}}$$ (11)
  *Algorithm process type of NDL*:
   if (T_4i_ > T_5i_) (*then ω*_*i*_ *= 1* and *sends to Cloud*)
   otherwise (*ω*_*i*_ *= 0* and *sends to Q*_*3*_)
*END*

As mentioned above, in the worst case, the complexity of a request is θ(1) because the SDN controller decides where to process the request based on its type. The most challenging decision-making situation occurs when the request is delay-sensitive. In these cases, the request is either processed in the local Fog node with a complexity of θ(1), sent to the Cloud with a check complexity of θ(1), or sent to neighboring Fog nodes with the check complexity depending on the number of neighboring Fog nodes (k). Since the number of neighbors of a Fog node is countable and constant, the complexity is also θ(1). If all the requests sent by an MTC machine are considered, the complexity depends on the number of requests.

VRs are a key component of Cloud computing, and they can also be used in an FC environment with the same efficiency. VRs can be used to create load-balanced environments, distributing traffic across multiple VRs to prevent overload and ensure high performance. Determining the number of VRs deployed on a resource is important. The number of VRs can be managed by SDN controller to balance the load, and the method of control is as follows:

1. Estimate response time by analyzing the header.
2. Monitor resources and their queues.
3. Check the performance of current VRs
4. Update the count of VRs by the SDN controller if needed

- If the count of flows on the VRs is high, and some of the delay-sensitive flows or more than half are rejected, then the count of VRs should be increased in the next period.
- If the maximum efficiency of the VRs is not being utilized, their number should be reduced in the next period. In other words, some of them should be turned off to save energy.
- If the arrival rate of flows to the Fogs is almost constant after a few periods, the count of VRs in different periods becomes almost the same. If the count of VRs is appropriate, the same number will be used for the next period.

The following algorithm provides a concise and comprehensive outline of the proposed method.

*For (i = 1 to r)*

  *{Sending requests to the main SDN controller and identifying its type*

   *If (type of request = = DL or DH) then send to Q*_*1*_ *(first queue of edge switches)*

   *Else if (type of request = = NDL) then send to Q*_*2*_ *(second queue of edge switches)*

  *Run polling algorithm in OpenFlow edge switch and send to Fog layer*

 *If (DL){*

 *If (T*_*QRi*_ *< T*_*2i*_*){(then ζ*_*i*_ *= δ*_*i*_ *= θ*_*i*_ *= 0 and send to Q*_*1*_*)*

 *Else (for j = 1*:*n_n if (T*_*nj*_ *< T*_*2i*_*) (then ζ*_*i*_ *= 1 and δ*_*i*_ *= θ*_*i*_ *= 0 and send to Fog neighbor j)*

 *Else if (T*_*ci*_ *< T*_*2i*_*) (then δ*_*i*_ *= 1 and θ*_*i*_ *= 0 and send to Cloud)*

 *Else (θ*_*i*_ *= 1 and reject)}*

 *Else if (DH){*

 *If (T*_*ci*_ *< T*_*2i*_*) {(then δ*_*i*_ *= 1 and θ*_*i*_ *= 0 and send to Cloud)*

 *Else if (T*_*QRi*_ *< T*_*2i*_*) (then ζ*_*i*_ *= δ*_*i*_ *= θ*_*i*_ *= 0 and send to Q*_*1*_*)*

 *Else (for j = 1*:*n_n if (T*_*nj*_ *< T*_*2*_*) (then ζ*_*i*_ *= 1 and δ*_*i*_ *= θ*_*i*_ *= 0 and send to Fog neighbor j))*

 *Else (θ*_*i*_ *= 1 and reject)}*

 *Else if (NDL){*

 *Calculation of* Eq 11

 *If (T*_*4i*_ *> T*_*5i*_*) (then ω*_*i*_ *= 1 and sends to Cloud)*

 *Else (ω*_*i*_ *= 0 and sends to Q*_*3*_*)*

 *Else if (NDH) then sends to Cloud}*

 *Run polling algorithm in VRs*

 ***}***

 ***}***

### 3.3. Queueing model and scheduling

The flows received for multiple queues of different priority processes are shown in Fig 4. Each queue is empty and idle when t < 0. Each resource queue follows a birth-death process or M/M/1 model, which can be extended to the M/M/m and M/M/k model. The First-Come-First-Serve (FCFS) queueing model serves the flows in the same queue, where m and k indicate the count of active VRs in the Fog layer or the number of switches in the edge.

In the proposed model, queueing theory is required in two cases to calculate the queueing delay. The first case is when a request is sent from MTC layer to Fog layer through OpenFlow edge switches (*T*_*QS*_), and the second case is when flows are waiting for processing by VRs in the Fog layer (*T*_*QR*_) (The equations are written only for the M/M/m model. By replacing k with m, *T*_*QS*_ can also be obtained). A Poisson process models the incoming flows of arrival rate realistically; the time interval between the consecutive flow arrivals is exponentially distributed. Let *λ* and *μ* represent the arrival rate and the service rate (or VRs’s potential to serve flows per time) of the resources, respectively. Furthermore, the arrival rate and service rate are the same as *λ*_*h*_ *= λ*, where *h = 0*,*1*,*2*,*3*,*…*,*m*, and *μ*_*h*_ *= μ* where *h = 0*,*1*,*2*,*3*,*…*,*m*. The service rate is calculated from Eq (12) according to [53].


$$\mu_{\text{h}}=\text{min}\left[\text{h}\mu ,\text{m}\mu \right]=\left\{\begin{array}{rr} h\mu & 0\leq h\leq m\\ m\mu & h\leq \;m \end{array}\right.$$ (12)

In the proposed model, the average time that a flow spends in the system varies according to changes in the incoming flow rate. This is an important factor to consider when designing the system’s performance. The probability of all computing resources being idle is defined as follows, and is expressed as p_0_ in the Eq (13) according to [53].


$${\text{p}}_{0}={\left[1+\sum_{h=1}^{m-1}\frac{{\left(m\rho \right)}^{h}}{h!}+\sum_{h=1}^{\infty }\frac{{\left(m\rho \right)}^{h}}{h!}\;\frac{1}{m^{h-m}}\right]}^{-1}$$ (13)

The condition $\rho =\frac{\lambda }{\mu \;m}\leq 1$ applies to the probability (*p*_*h*_) that these coefficients satisfy, which is $p_{h}=p_{0}{\left(\frac{\lambda }{\mu }\right)}^{h}$, where *h ≥ 0*. *p*_*h*_ represents the probability that the *h*th computing resource is empty. The average time a flow spends waiting in the queue is calculated as *T*_*QR*_ and is more explicitly expressed in Eq (14) according to [53]. The QoS requirement of the MTC requests is guaranteed by Eq (15) according to [53]. If Eq (15) is satisfied, *T*_*QR*_ is optimal.


$${\text{T}}_{\text{QR}}=\frac{1}{\lambda }(\text{m}\;\rho +\rho \frac{{\left(\text{m}\;\rho \right)}^{\text{m}}}{\text{m}!}+\frac{{\text{p}}_{0}}{{\left(1-\rho \right)}^{2}})=\frac{1}{\mu }+\frac{{\left(\frac{\lambda }{\mu }\right)}^{\text{m}}}{\lambda \text{m}!}+\frac{{\text{p}}_{0}}{{\left(1-\frac{\lambda }{\text{m}\mu }\right)}^{2}}$$ (14)

$${\text{T}}_{\text{QR}}\leq \frac{1}{\mu -\lambda }$$ (15)

Without loss of generality, this article assumes that a queue with a larger number has a lower priority than a queue with a smaller number. The polling algorithm is non-preemptive for priority queues and is preemptive for non-priority queues [30, 54]. A parameter *0 < P*_*i*_ *≤* is assigned to each queue, where *I ϵ 1*, *2*, *3*, and *P*_*3*_ *= 1*. The SDN controller first polls queue 1 in each process flow. The flows at the top of the queue will be served with a probability of *P*_*1*_ When it is polled. The next non-empty queue is polled by the SDN controller with probability of *P*_*2*_*(1-P*_*1*_*)*, and the last queue is polled with probability *P*_*3*_*(1-P*_*2*_*)(1-P*_*1*_*)* [29]. The probabilistic priority queues are shown in Fig 4. If queue i = 1 is empty when it is polled, the SDN controller will not serve it and will move on to poll the next queue i+1. After serving a flow, the SDN controller starts polling queue one again. The normalized relative weight *r*_*i*_ of queue i is defined as Eq (16) according to [29]. Based on all non-empty queues’ normalized relative weights, at each stage, the probability *P*_*i*_ is expressed in Eq (17) according to [29].


$${\text{r}}_{\text{i}}={\text{P}}_{\text{i}}\prod_{\text{j}=1}^{\text{i}-1}(1-{\text{P}}_{\text{j}}),\sum_{\text{i}=1}^{3}\;{\text{r}}_{\text{i}}=1$$ (16)

$${\text{P}}_{\text{i}}=\frac{{\text{r}}_{\text{i}}}{1-\sum_{\text{j}=1}^{\text{i}-1}\;{\text{r}}_{\text{j}}}$$ (17)

The following steps generally indicate a simple implementation of the priority polling queues:

1. Calculate the relative weights for each queue based on Eq (16).
2. Finding the first non-empty queue. If all queues are empty, monitor all queues again, then calculate *sum*_*1*_ *= r*_*1*_, *sum*_*2*_ *= r*_*2*_ *+ r*_*1*_ and *sum*_*3*_ *= r*_*3*_*+ r*_*2*_ *+ r*_*1*_.
3. Obtain a random number *0 ≤ RN ≤ 1* uniformly distributed and find the first queue such that *RN≤sum*_*i*_. First, check Q_1_, then Q_2_, and finally Q_3_.
4. Serve the head flow of the selected queue, and then repeat the same steps until all the flows are served.

**Fig 5:**
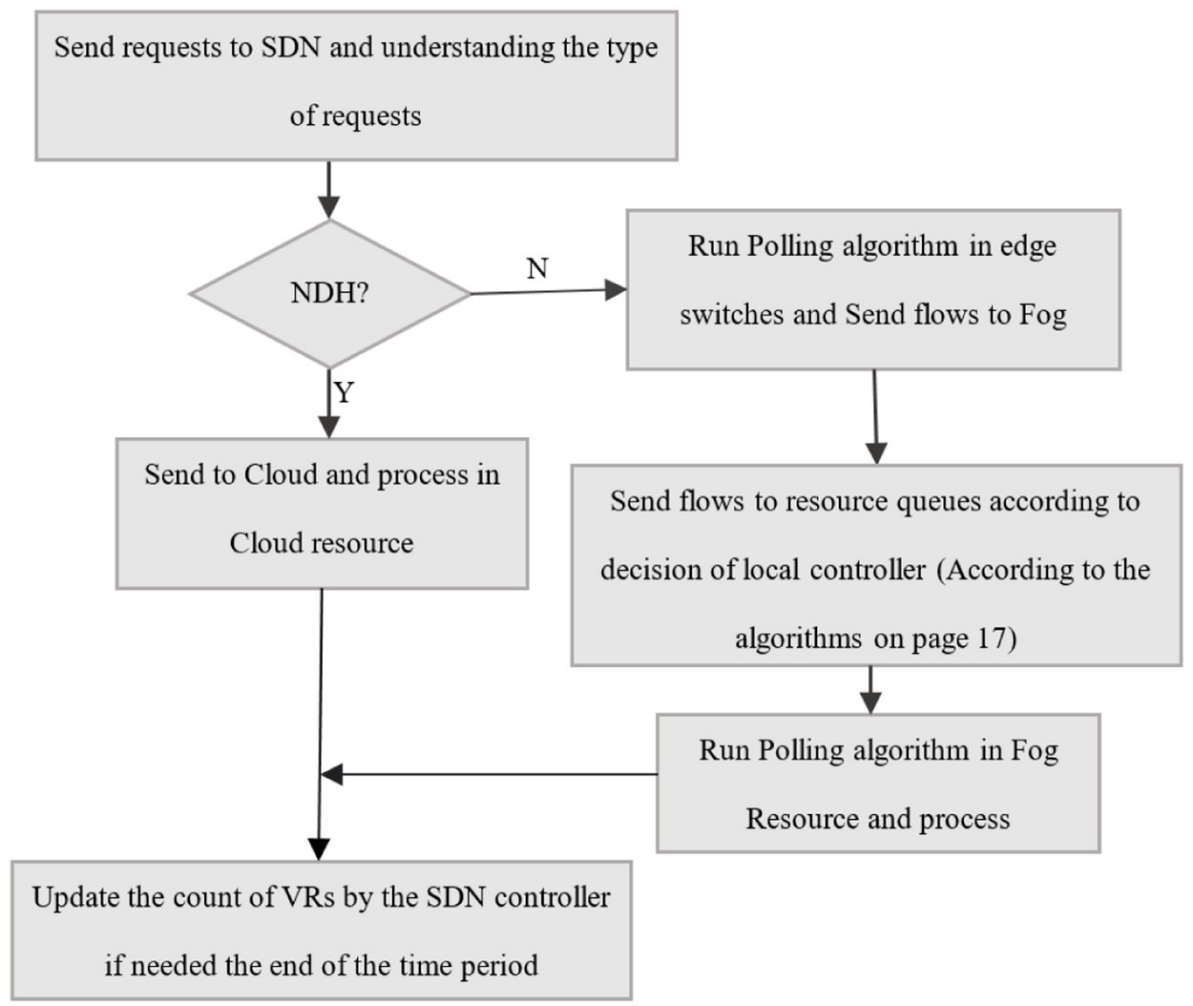
Policy of SDN controller for handling the forwarding requests.

The procedure of forwarding requests by the SDN controller is shown in Fig 5.

## 4. Simulation experiments

To illustrate the advantages of the proposed task offloading scheme, the efficiency of the offloading service in an SDN based FC framework is evaluated to minimize MTC total delay for different types of flows and to remove the starvation problems based on the schedule for lower priority flows. Simulation results are presented in this section to validate the total delay based on SDN virtualization in FC by workload allocation based on offloading policy. To investigate the performance, a simulation environment of the proposed task offloading scheme has been developed in Matlab on a desktop computer. This model has been implemented and examined in different experimental settings.

### 4.1. Simulation setup

The simulation area is assumed to be 200×200 m^2^, and a certain count of nodes are randomly distributed in the area. The scenario generally considers 500 MTC nodes and 500 to 10,000 requests sent from MTC machines, four Fog resource nodes, three OpenFlow switches, and Cloud servers in the proposed system. Initially, one VR is placed in each Fog node, and this number is increased to improve efficiency. The number of VRs in each Fog varies optimally according to the number of flows. The simulation can be extended to include more Fog devices and more MTC nodes, with similar results. The MTC node sends its flow to Cloud servers or Fog resources. Flows sent to the Fog layer are processed based on the proposed system in local Fog resources, to neighbor Fog resources, or the Cloud servers. MTC nodes have a small propagation delay with the Fog because they are at a small distance (can be ignored).

In order to manage the MTC network using an SDN controller, the network needs to be set up first. Then, the SDN controller can be implemented using the Matlab SDN Toolbox. Afterward, the SDN controller can be connected to the MTC network by configuring the switches to forward traffic to the controller. Additionally, the Parallel Computing Toolbox in MATLAB is utilized to implement Cloud computing services. This tool allows users to run their computations in parallel across multiple systems, either locally or on Cloud platforms, and facilitates the harnessing of computing resources at scale.

VRs are software emulations of physical machines. To implement VRs, a selection of virtualization software such as VMware needs to be made initially. Then, virtual networks and VRs need to be set up and allocated. Finally, to simulate the proposed network model designed by Matlab and develop it, the virtual networks between the VRs should be set up.

A processing flow length of 100 bytes is assumed for light flows, and 1 MB for heavy flows on average. This proposed scheme assumes that the processing speed rate in the Cloud server is 100 times faster than a Fog resource on average. It is also assumed that the average processing time of the MTC node in a Fog resource is 25 ms and 425 ms for light flows and heavy flows, respectively. To take into account the deadline of flows, two different flow types are considered, including deadline-based flows and flows without deadlines, as deadline-based flows have different deadlines. The proposed design simulation was replicated 10 times for the generated random data. The maximum transmission bandwidth between the MTC machines and the Fog is 100 Mbps, and the maximum transmission bandwidth between the Fog and the Cloud is 10Gbps. Some simulation parameters are defined according to [4]. Table 4 summarizes other simulation parameters (~ means about).

**Table 4:** Simulation parameters.

parameter	value
simulation area	200×200m
λ	~27
μ	~40
Bandwidth Fog	100Mbps
Bandwidth Cloud	10Gbps
Distance Fog	400m
Distance Cloud	500km
OpenFlow version	1.3
Number of requests	1000 to 10000
light flows length and heavy flows length	~100 bytes and ~1 MB
average processing time for Light and Heavy flows	~25 ms and ~425 ms
The number of scenarios repetitions	10

**Fig 6:**
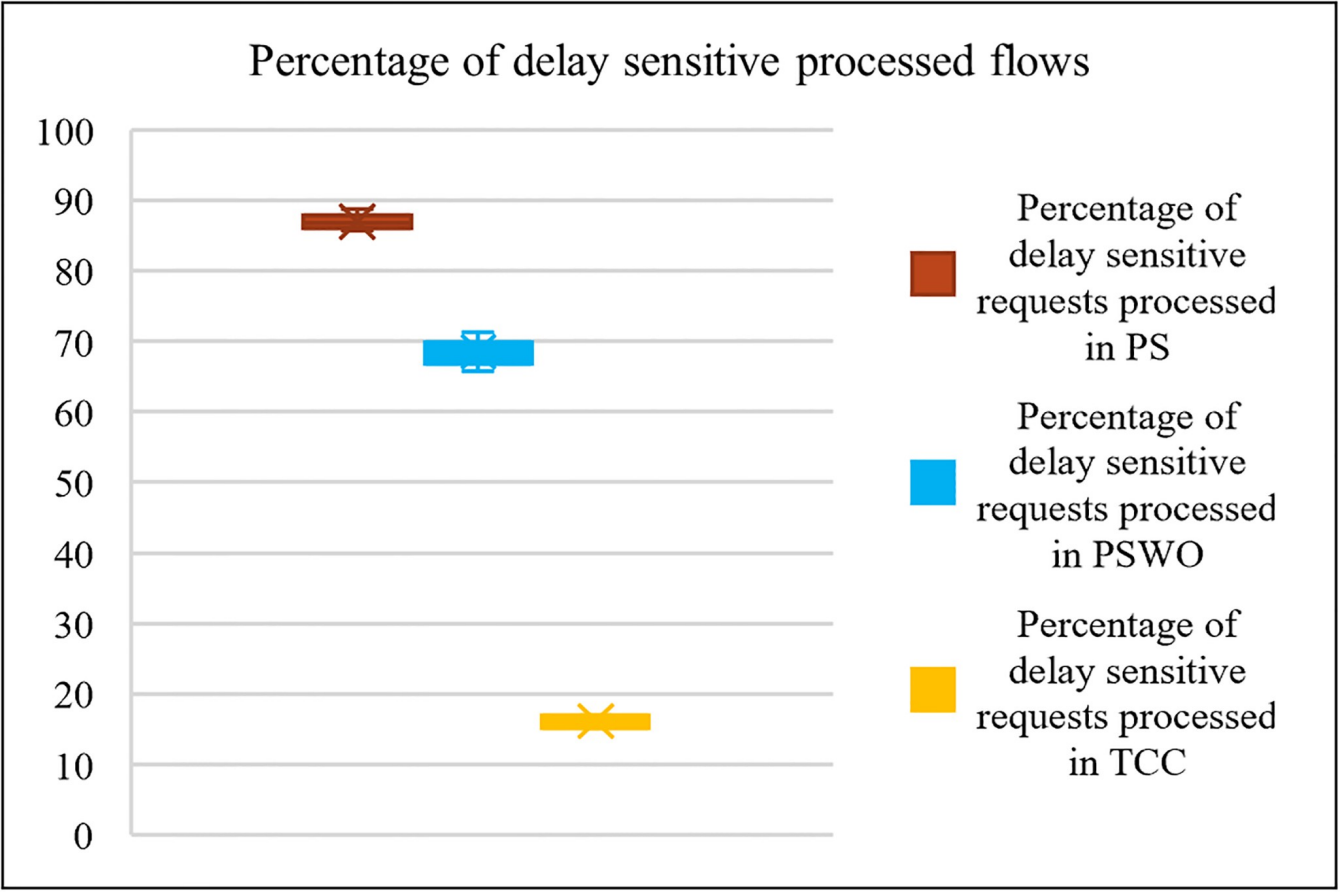
Comparison the percentage of delay sensitive processed flows.

**Fig 7:**
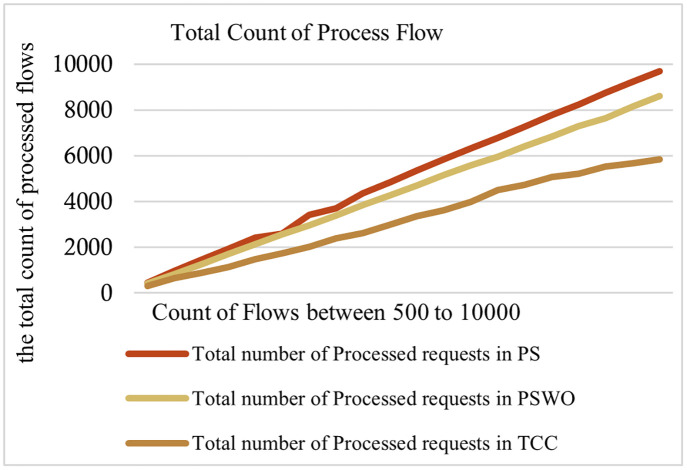
Comparison of the total count of processed flows.

**Fig 8:**
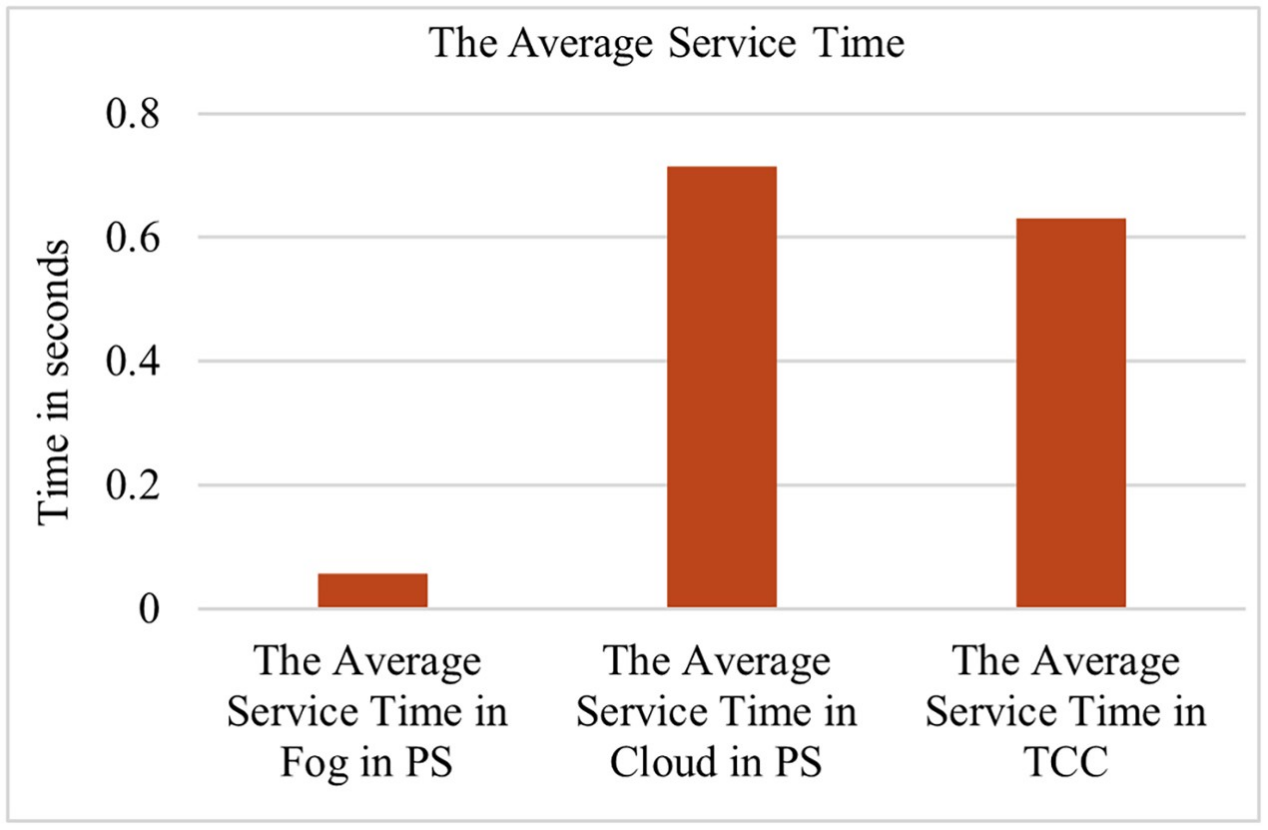
Comparison the average service time.

**Fig 9:**
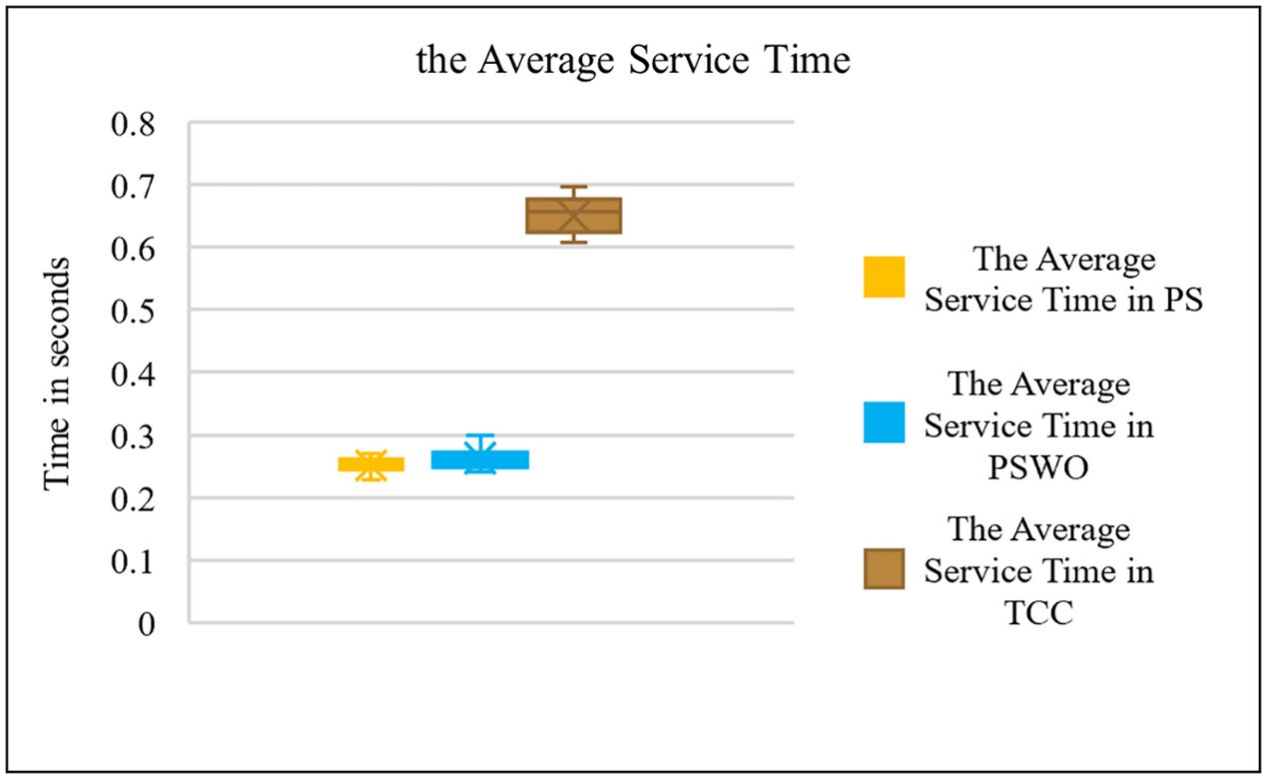
Comparison the average service time.

**Fig 10:**
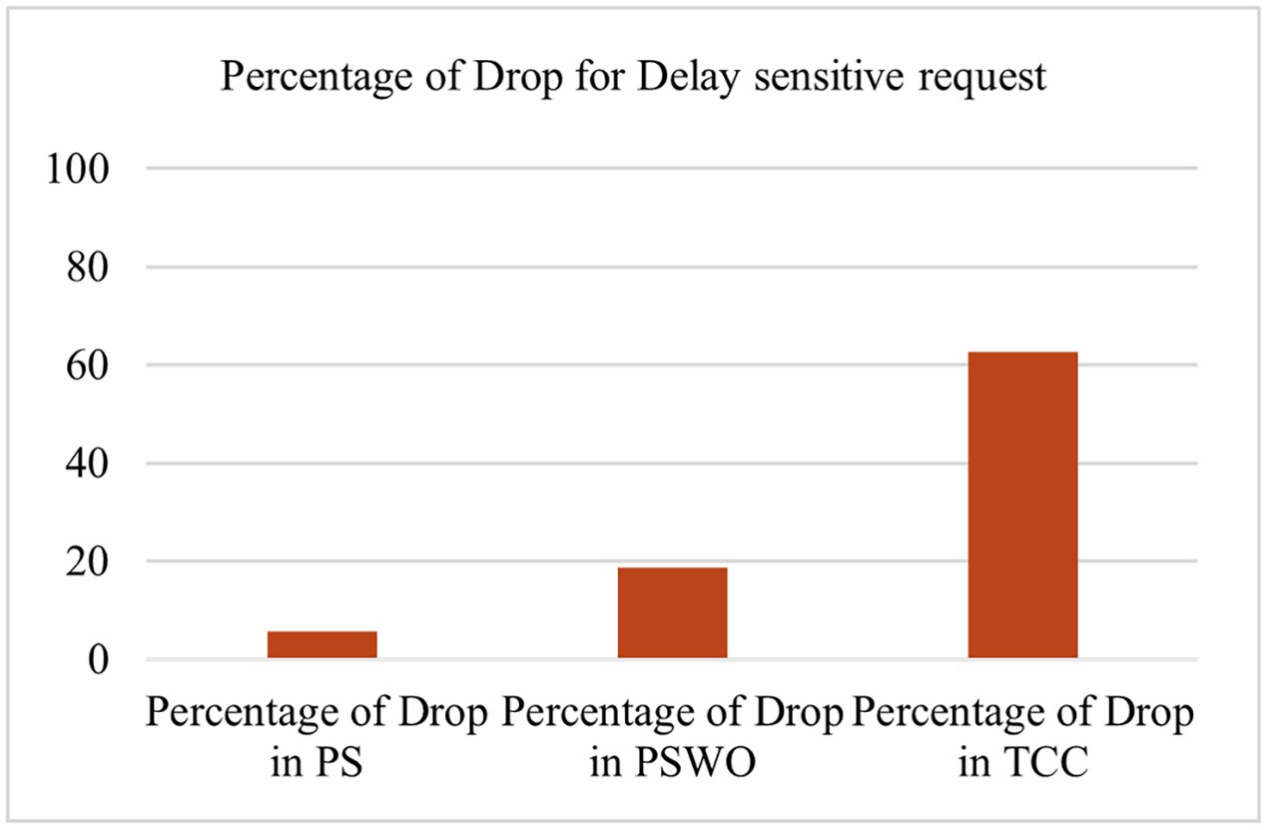
Comparison the percentage of drop for delay sensitive flows.

**Fig 11:**
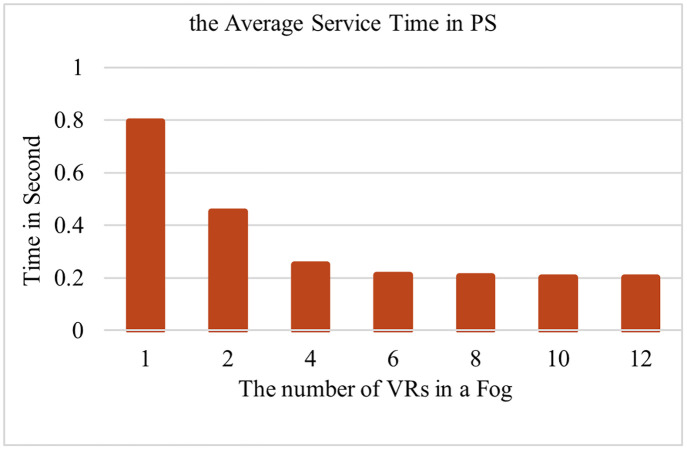
Impact of the average service time with variable VRs.

**Fig 12:**
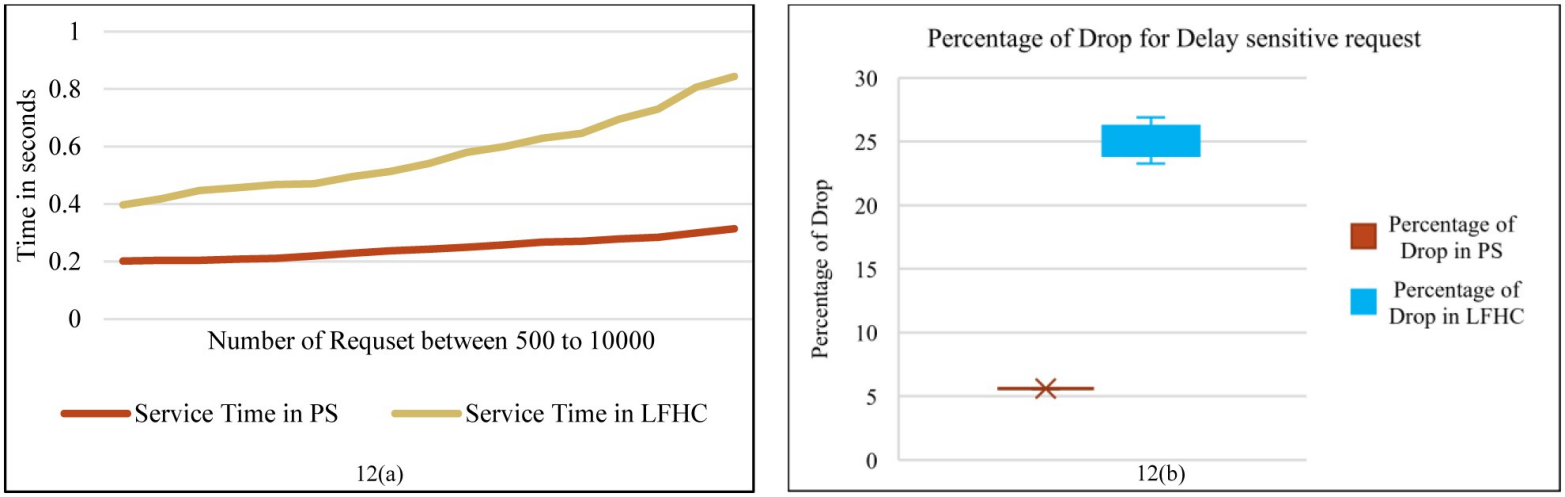
Comparison the average service time and percentage of drop in PS & LFHC.

**Fig 13:**
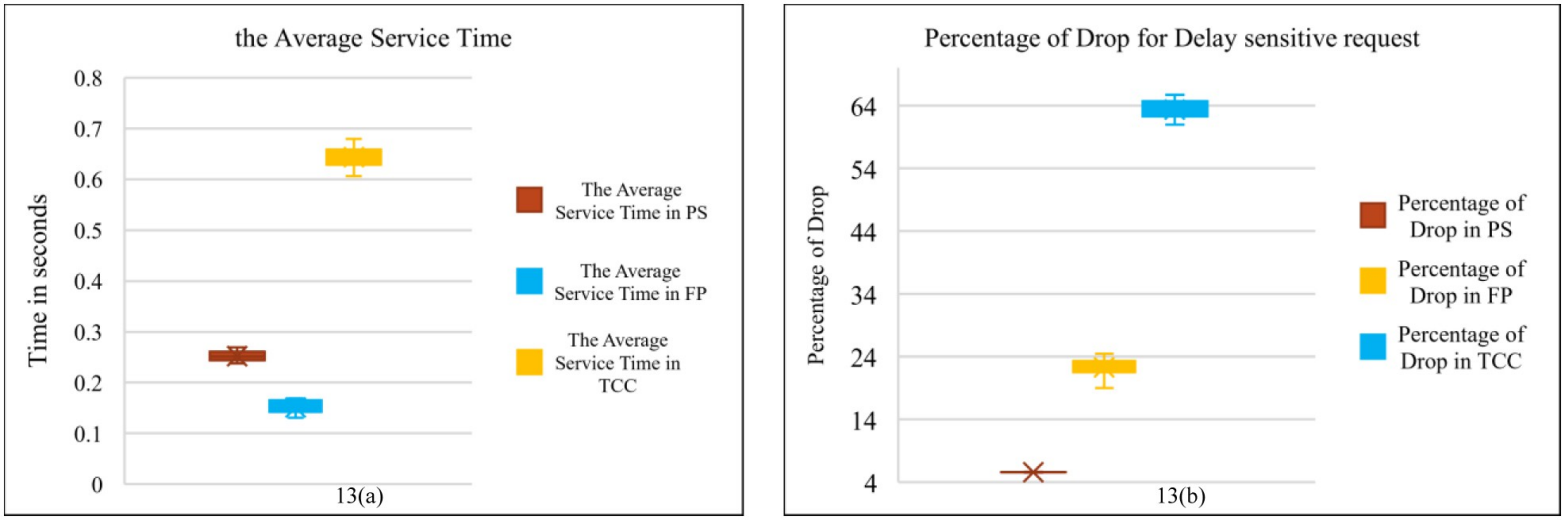
Comparison average service delays and percentage of drop in PS & FP & TCC.

**Fig 14:**
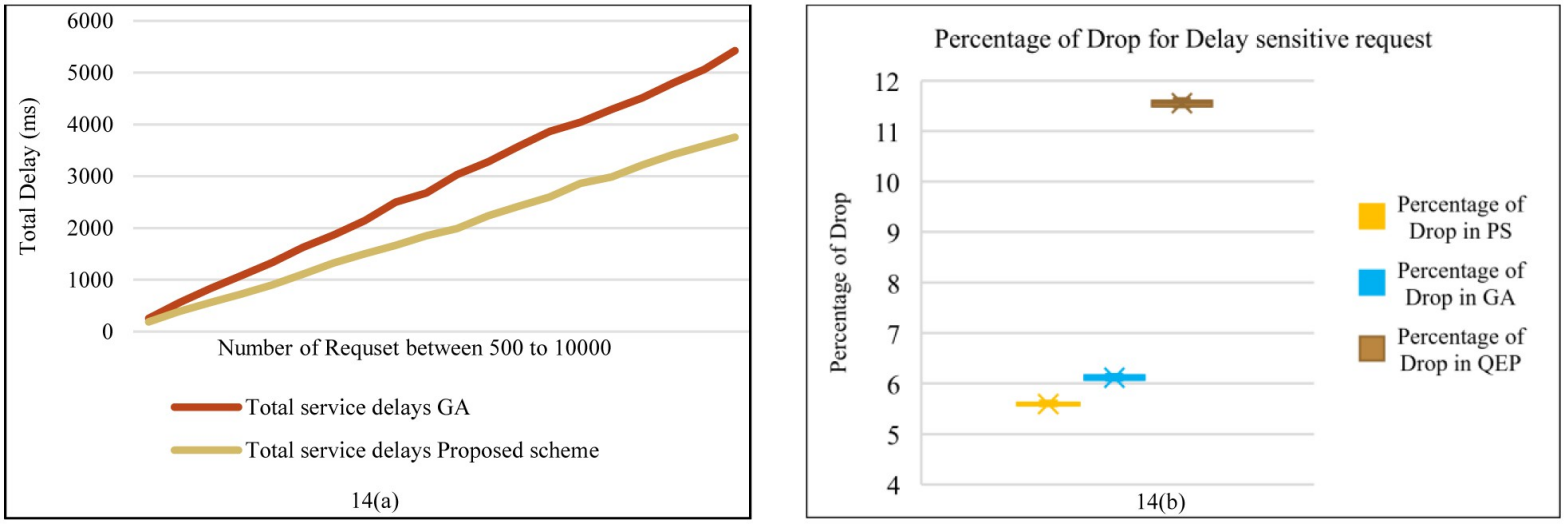
**(a)**. Comparison service delays in proposed scheme & GA for non-delay-sensitive flows. **(b)**. Comparison the percentage of drop in PS & QEP & GA.

**Fig 15:**
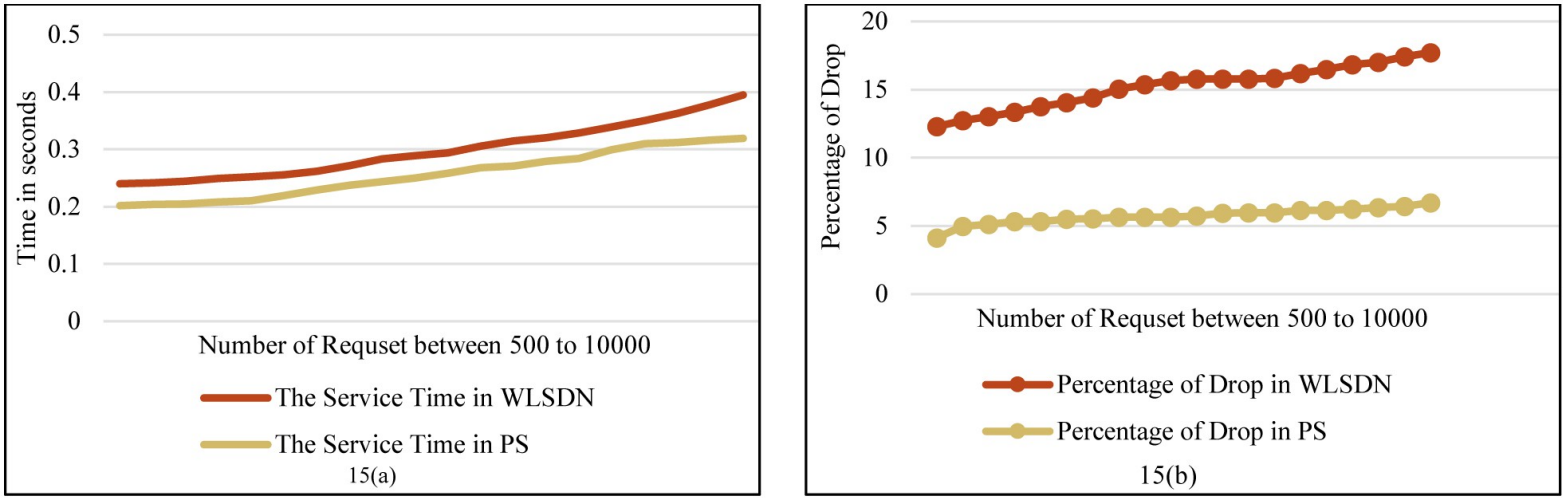
**(a)**. Comparison service delays in proposed scheme & GA for non-delay-sensitive flows. **(b)**. Comparison the percentage of drop in PS & WLSDN.

**Fig 16:**
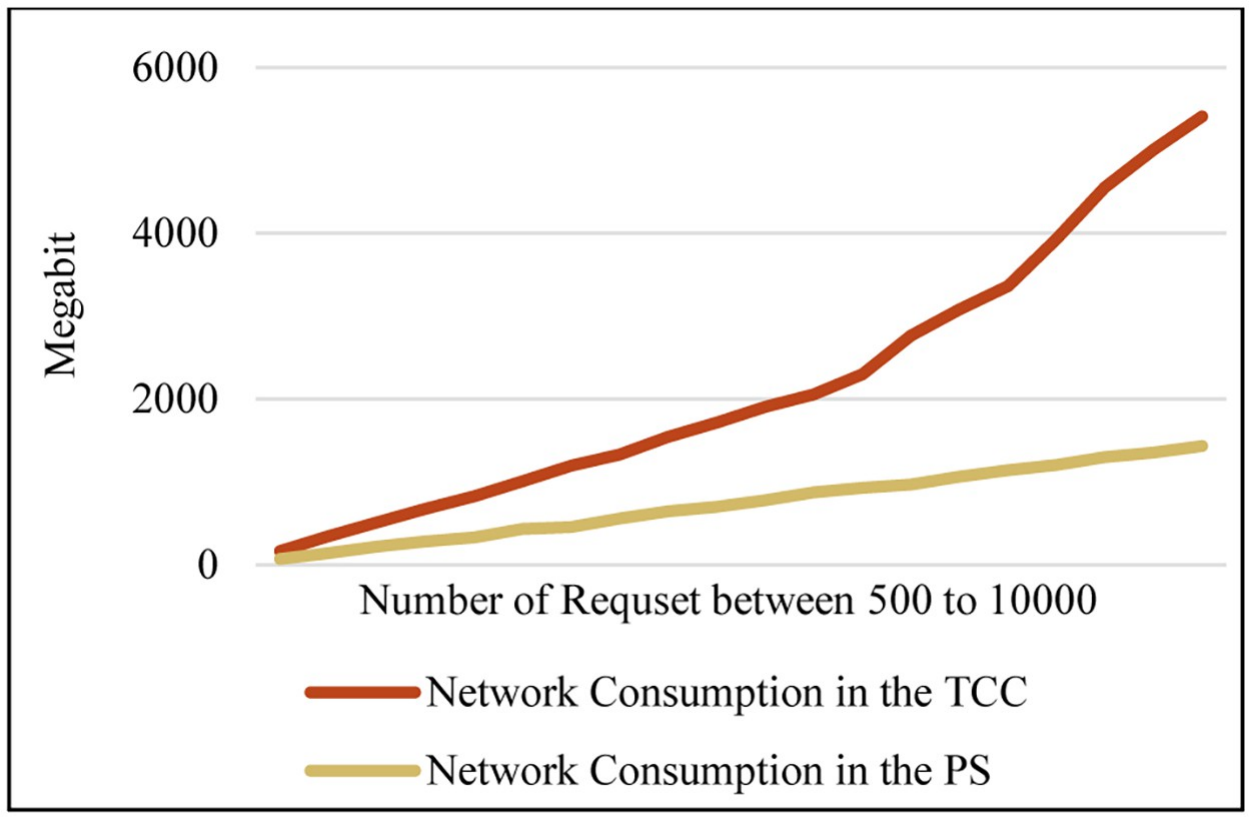
Comparison network consumption.

**Fig 17:**
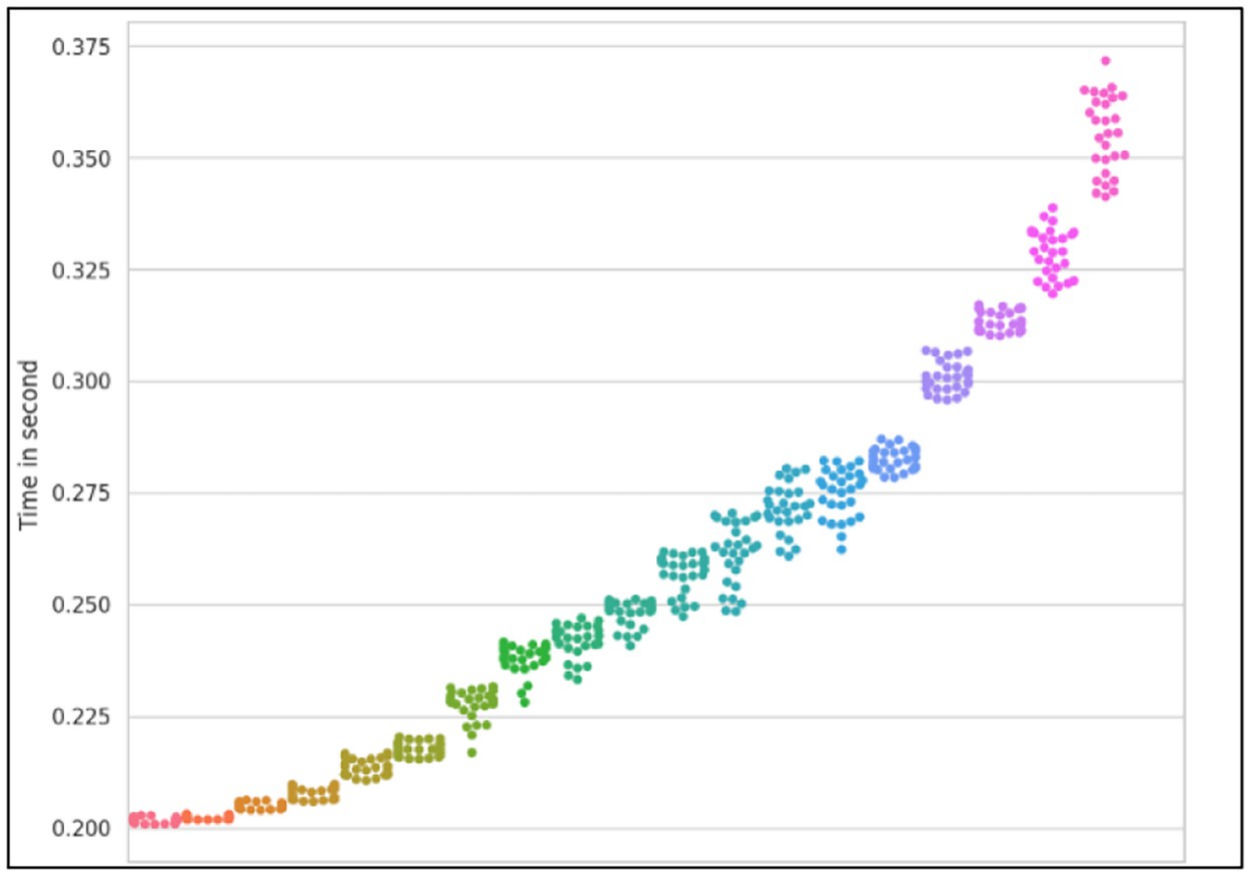
Average service delays in proposed scheme using the swarm plots.

**Fig 18:**
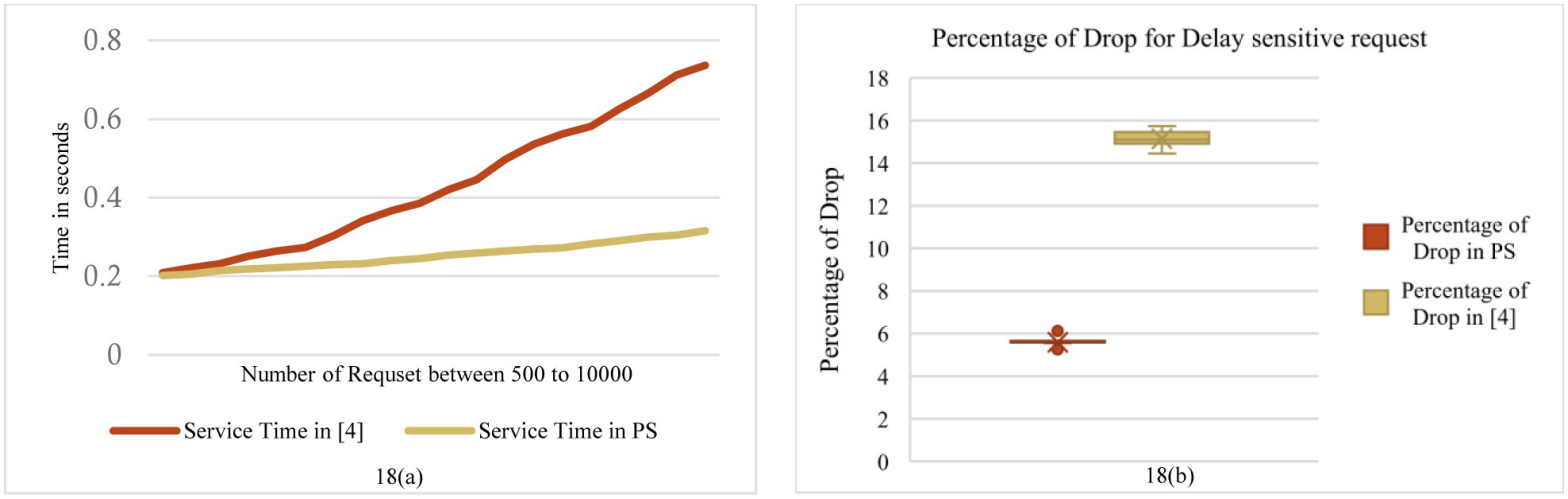
**(a)**. Comparison the Average service time in PS & The proposed method in article [4]. **(b)**. Comparison the percentage of drop in PS & [4].

To understand the benefits of the proposed hierarchical architecture, the performance of the proposed approach has been compared and evaluated with the following traditional approaches in terms of the average service delay, percentage of drop for delay-sensitive flows, network consumption, the total count of processed flows in three modes, the percentage of offloading flows, and several other parameters to examine the efficiency of the proposed method. The Proposed Scheme is labeled as PS, while one mode is Proposed Scheme Without Offloading labeled as PSWO and Traditional Cloud Computing labeled as TCC. These TCC act as the main approach to illustrate the efficiency development of the PS in the Fog offloading strategy. These parameters are shown in Figs 6–18.

### 4.2. Numerical results

Fig 6 illustrates the percentage of delay-sensitive processed flows in PS, PSWO, and TCC. This metric answers the question of what percentage of delay-sensitive flows are processed and what is the impact of the proposed method on delay-sensitive flows. This chart only focuses on the DL and DH types of flows. In other words, only the first and second parts of the algorithm presented on page 18 have been evaluated. As shown in Fig 6, the percentage of delay-sensitive processed flows in PS is about 21% better than PSWO and about 80% better than TCC. TCC processes the flows regardless of their priorities and deadlines, which decreases the processed flows. Consequently, the simulation results show that PS performs better than PSWO and TCC. Fig 7 illustrates the total count of processed flows in three modes. This diagram is one of the most important diagrams obtained in the simulation section. To draw it, all the equations and parts of the proposed method in the previous section have been used. In summary, all the steps of the flowchart in Fig 5 have been examined. Here, 20 different scenarios are considered while varying the counts of flows. As shown in Fig 7, the total count of processed flows is about 96.58% in PS, 85.46% in PSWO and 58.53% in TCC. As shown in Fig 7, the total count of processed flows of PS is higher than PSWO and TCC, because PS process higher priority flows faster than the other flows; therefore, the count of dropped flows decreases. In the proposed method, the Fog mechanism and offloading delay-sensitive flows to the neighboring Fogs use increasing service flows.

The significant aim of the FC system is to reduce the average service time for MTC requests, when the number of requests changes from 500 to 10000, which is the a crucial requirement for real-time requests. Diagrams 8 and 9 calculate the average service time. As the title of the article suggests, the main goal of this study is to reduce the delay. Therefore, after identifying and processing a flow using the proposed method, the total delay of requests sent from MTC layer is obtained in the Cloud or Fog layer. Figs 8 and 9 compare the average service time in Fog and Cloud for PS, PSWO, and TCC (Service delay refers to the time it takes to process an MTC flow when an MTC machine forwards a flow until it receives the response for that flow). As shown in Fig 8, the average service time in Fog in PS is about 57ms and in Cloud in PS, it is about 710ms. As shown in Fig 9, both PS and PSWO have significantly reduced the average service time by about 60%. It is observed that the flow service time in the Cloud in PS is longer than in TCC because in PS, heavy flows are mostly sent to the Cloud, while in TCC, both heavy and light flows are sent to the Cloud.

One important criterion of the proposed solution is the percentage of dropped delay-sensitive MTC flows. Fig 10 illustrates another simulation to determine the percentage of dropped delay-sensitive MTC flows for PS, PSWO, and TCC. The count of flows with different delay-sensitive categories meets their different respective deadlines. As shown in Fig 10, the count of drops in PS has decreased significantly, regarding PS it is 5.6%, regarding PSWO it is 32.2%, and regarding TCC, it is 62.6%. It should be noted that the percentage of offloading flows in PS is 7.59%. Fig 11 illustrates another simulation to determine the variable VRs in PS. This graph investigates the effects of changing the number of VRs on delay calculation. This graph pertains to the final part of subsection 3.2. By making the same requests from the MTC layer with a different number of VRs, the optimal number of VRs is obtained. In other diagrams obtained in this section, the number of VRs is initially set to the optimal value obtained in this section, and it is changed if necessary. The simulation is repeated with 7 different values for VRs in a Fog. It illustrates the comparison of the processing flows while the count of flows is kept constant at 10000. First, m = 1 is considered in each Fog, then the count of VRs increases. As it can be seen, increasing the count of VRs to each Fog substantially decreases the average service delay due to offloading, resulting in the maximum performance. On the other hand, when VRs = 6 increases, it has a little effect on reducing the service delay time because the Fog resource processing power is finite.

According to Fig 12(a), the average service delay is compared between two modes of the proposed scheme. This figure illustrates the effect of processing location on service delay, comparing the proposed scheme’s service delay with the "LFHC" (Lightweight in Fog & Heavy in Cloud) mode where lightweight flows are processed in the Fog layer and heavy flows are processed in the Cloud layer. In the second mode, the queues behind Fog resources are reduced to two, one for delay-sensitive flows and one for non-delay-sensitive flows. The SDN controller sends all lightweight flows to the Fog layer without making any decision on the processing location. It also sends DH flows to the Cloud layer only if there is still a processing deadline, without making any decisions about their processing location. Thus, that part of the algorithm that deals the processing location is removed. The average delay in both modes is almost the same, but the percentage of dropped delay-sensitive requests is lower in the proposed scheme, as shown in Fig 12(b).

Fig 13(a) shows another simulation to determine the average service delay in three modes when the number of requests changes from 500 to 10000. This figure shows the effects of processing location on the service delay when all requests are lightweight. The first mode is the proposed scheme, while the second mode is when all requests are sent directly to the Fog layer labeled as FP (Fog Processing). The third mode is when all requests are sent directly to the Cloud layer. As the figure shows, the average service time in the first mode gets closer to that of the second mode, but the percentage of dropped requests is lower in the proposed scheme, as shown in Fig 13(b).

Fig 14 shows the percentage of dropped flows in another simulation of the resource queue priority. In other words, these figures show the effects of changing the priority of the queues in the resource Fog layer and edge switches on the percentage of dropped flows, compared in three modes. The first mode is the proposed scheme. In the second mode, all resource queues in the Fog layer have equal priority, labeled as QEP (Queues with Equal Priority). In this scenario, only one queue is formed behind OpenFlow edge switches and Fog resources, and all flows are placed in it and processed FCFS method. The SDN controller decides whether to send flows to the Cloud layer or the Fog layer based on the proposed algorithm, avoiding multiple queues and the starvation problem for requests in lower priority queues. In the third case, the queue of resources is prioritized, which is a greedy algorithm. The greedy algorithm is defined for queues without considering polling algorithms. It is a heuristic) model for solving a problem by making the available optimal choice at every step. It does not provide a globally optimal solution, but can produce local optimization [55]. The third mode is labeled as GA (Greedy Algorithm). This scenario is similar to the proposed algorithm, but the final part of subsection 3.3, which deals with solving the starvation problem using the polling algorithm, is removed. Fig 14(b) shows that the percentage of dropped flows increases in QEP, but in the first and second modes, it is almost equal. However, the starvation problem of non-delay-sensitive flows increases in the greedy algorithm.

Fig 15 shows the service delay and the percentage of dropped flows as another simulation of the local SDN controller. In other words, this figure shows the effects of removing the local SDN controller in the Fog layer. The percentage of dropped flows is compared in two modes. The first mode is the proposed scheme. In the second mode, the local SDN controller is removed in the Fog layer, labeled as WLSDN (Without Local SDN controller). In the second mode, the decision to offload flows to neighboring Fog nodes is made using the main controller. In other words, in this scenario there is only one main and central SDN controller. Therefore, in addition to the proposed model delay, the round-trip time to the SDN controller must also be added to it. Furthermore, given that the number of requests towards the main SDN controller is nearly doubled, the processing duration within the SDN controller correspondingly increases. As the Fig 15(a), the average service time in the PS gets closer to that of WLSDN. As the Fig 15(b) shows, the percentage of dropped flows increases in the WLSDN mode.

Reducing network consumption is another effect that has been compared in the PS and the TCC. Network consumption means the number of transmitted bits to the network core, which is obtained by multiplying the number of requests sent from the MTC layer to the Cloud layer for processing by the number of bits in each request, and then summing them up. Fig 16 illustrates that the PS has improved by about 65% compared to TCC. In Fig 17, the service delay in the proposed method with more details is shown using a swarm graph. In this graph, the number of requests varies between 500 and 10000. This scenario has been repeated approximately 30 times. The x axis indicates an increase in the number of requests from left to right. As the chart indicates, the average service time does not change significantly with an increase in the number of requests.

The last graph in Fig 18 illustrates the comparison between the proposed method and the method presented in [4]. One of the most significant differences between this article and the proposed method is the absence of a controller. In the approach presented in [4], in order to check the processing of requests, the requests must be sent to the local Fog node to check the available resources for processing. If the local Fog node is unable to process the request, it will be offloaded in a serial manner to neighboring Fog nodes. Each neighboring node checks whether it is able to process the request or not. If all the neighboring nodes are unable to process the request, it is sent to the Cloud. Fig 18 shows the average service delay and the percentage of dropped flows, which performs better in both cases of the proposed method.

Finally, in this article, a statistical method has been used to validate the proposed method. For this purpose, the t-test statistical method has been chosen. The t-test is a statistical test used to determine whether the means of two groups are significantly different from each other. It is a powerful statistical tool that is widely used in various fields. The proposed method’s superiority over other defined scenarios was demonstrated in Figs 6 to 18. The t-test is used to determine whether this difference is significant, i.e., whether the proposed method is valuable or not. Table 5 displays the results of comparison of the average service time in the PS with other scenarios. Table 6 displays the results of comparison percentage of drop in the PS with other scenarios. In these table, “✓” means a significant difference between two methods, and “✘” means no significant difference between two methods.

**Table 5:** Comparison of the average service time in the PS with other scenarios using t-test.

	TCC	PSWO	LFHC	FP	GA	QEP	WLSDN	[4]
PS	✓	✓	✓	✘	✘	✓	✓	✓

**Table 6:** Comparison percentage of drop in PS in the PS with other scenarios using t-test.

	TCC	PSWO	LFHC	FP	GA	QEP	WLSDN	[4]
PS	✓	✓	✓	✓	✓	✓	✓	✓

## 5. Conclusion

This article proposed a novel optimization offloading method for MTC communications in SDN and Fog. The method employed differential and priority-driven flow space allocation for heterogeneous MTC machines per-flow classes to satisfy their priority. The delay-sensitive flows were assigned to a configuration of priority queues on each Fog. In the proposed framework, an SDN-based load balancing approach for FC through offloading F was explained. The SDN controller’s global network knowledge was applied to make optimal decisions regarding task offloading. Polling algorithms can be applied to service flows according to queueing theory for resources. Simulation outcomes illustrated that, on average, the percentage of dropped flow for the proposed method and Cloud processing was 5% and 34%, respectively. Moreover, simulation results revealed an improvement in the average service time, the percentage of delay-sensitive processed flows, and the network consumption in the proposed method by about 60%, 80%, and 65% compared to traditional Cloud computing, respectively.

An interesting research topic for the future is to incorporate power consumption costs and deployment with the user’s mobility into the proposed scheme. Designing analytical models to optimize the offloading method is one of the proposed scheme’s next goals. Future challenges include optimizing route selection in the Fog layer and implementing the proposed method in VANETs and the healthcare system.
